# Bimetallic Ru–Pd Nanoparticles Anchored on Carbon Nanotubes for High-Performance Electrochemical Detection of Tryptophan: Experimental and DFT Insights

**DOI:** 10.1021/acsomega.6c02081

**Published:** 2026-08-10

**Authors:** Ömrüye Özok Arıcı, Bassam A. Najri, Emrah Kavak, Aykut Caglar, Arif Kivrak, Hilal Kivrak

**Affiliations:** † Department of Biology, Faculty of Science, 53004 Eskisehir Osmangazi University, Eskisehir 26040, Turkey; ‡ Nanotechnology, Sensor, and Energy Materials (NanotechSEM) Research Laboratory, Eskisehir Osmangazi University, Eskisehir 26040, Turkey; § Department of Chemistry, Faculty of Science, Eskisehir Osmangazi University, Eskisehir 26040, Turkey; ∥ Science and Technology Research and Application Center (BİTAM), Necmettin Erbakan University, Konya 42090, Turkey; ⊥ Department of Basic Sciences, Faculty of Engineering, Architecture and Design, 162311 Bartin University, Bartin 74100, Turkey; # Department of Chemical Engineering, Faculty of Engineering and Architectural Sciences, Eskisehir Osmangazi University, Eskisehir 26040, Turkey; ∇ Department of Chemical Engineering, Faculty of Engineering, Kyrgyz-Turkish Manas University, Bishkek 720038, Kyrgyzstan

## Abstract

A highly sensitive electrochemical sensor for the determination of tryptophan was developed using a bimetallic ruthenium–palladium catalyst supported on carbon nanotubes (Ru–Pd/CNT). The Ru–Pd/CNT nanocomposite was synthesized via a sodium borohydride (NaBH_4_) reduction method, enabling the uniform deposition of Ru and Pd nanoparticles onto the CNT surface. Comprehensive physicochemical characterization using X-ray diffraction (XRD), scanning electron microscopy with energy-dispersive X-ray spectroscopy (SEM-EDX), inductively coupled plasma mass spectrometry (ICP-MS), transmission electron microscopy (TEM), X-ray photoelectron spectroscopy (XPS), and temperature-programmed techniques confirmed the successful formation of a well-dispersed bimetallic system with strong metal–support interactions and mixed oxidation states. The Ru–Pd/CNT catalyst was used to modify a glassy carbon electrode (Ru–Pd/CNT@GCE) and was evaluated as an electrochemical sensor for tryptophan using cyclic voltammetry (CV), differential pulse voltammetry (DPV), and electrochemical impedance spectroscopy (EIS). The modified electrode exhibited strong electrocatalytic activity toward tryptophan oxidation and provided a large linear dynamic range (LDR) of 1–1200 μM, a low limit of detection (LOD) of 0.05 μM, and a sensitivity of 0.0023 A M^–1^. Density functional theory (DFT) calculations provided theoretical insight into the enhanced sensing performance. Comparative DFT calculations for CNT, Pd/CNT, Ru/CNT, and Ru–Pd/CNT showed that the HOMO–LUMO energy gap followed the order CNT > Pd/CNT > Ru/CNT > Ru–Pd/CNT, decreasing from 7.498 eV for pristine CNT to 0.466 eV for Ru–Pd/CNT. This trend indicates enhanced electronic activation and improved charge-transfer potential in the bimetallic Ru–Pd/CNT system. Molecular electrostatic potential (MEP), electron localization function (ELF), and localized orbital locator (LOL) analyses also indicated favorable electron redistribution and balanced localization–delocalization behavior at the metal–CNT interface. Based on the combined experimental and theoretical results, the Ru–Pd/CNT@GCE sensor represents a promising platform for sensitive and selective tryptophan detection under controlled analytical conditions.

## Graphical abstract

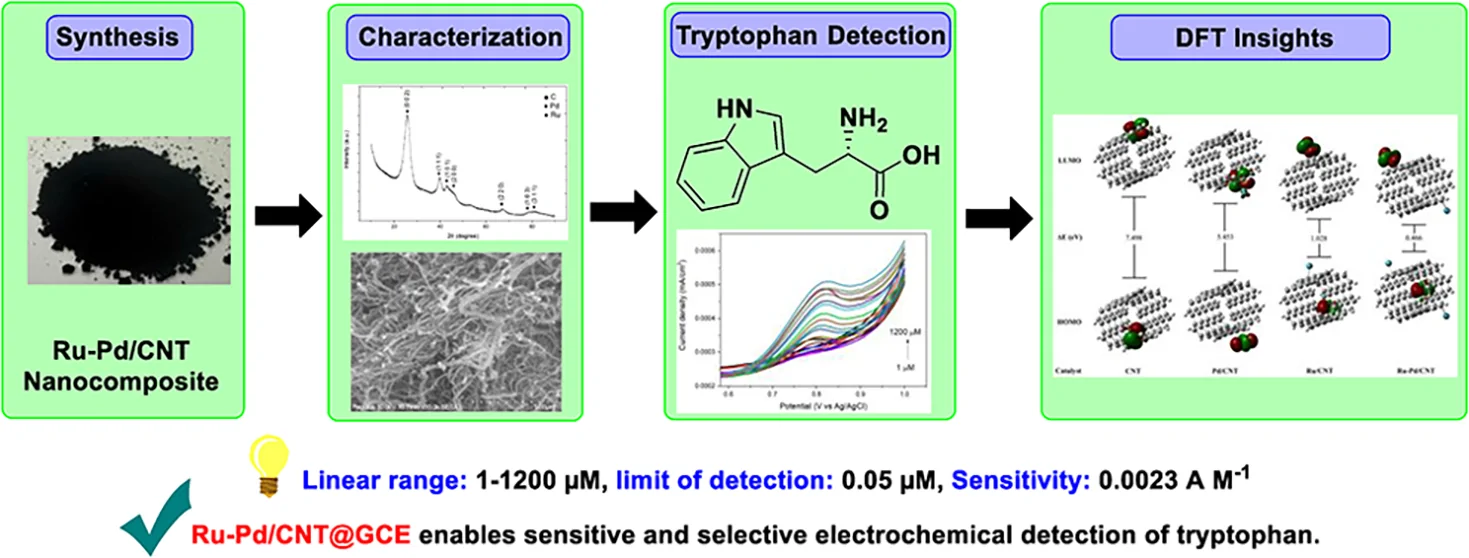

## 1 Introduction

Tryptophan is an essential aromatic amino acid that plays a vital role in several physiological and biochemical processes, including protein synthesis, production of serotonin and melatonin, immune homeostasis, and brain function.^1–3^ Excessive levels of tryptophan have been linked to a number of pathological conditions, including depression, neurodegenerative diseases, inflammation, and metabolic diseases.^4–7^ Therefore, the precise and sensitive determination of tryptophan is of great importance in clinical diagnostics, pharmaceutical analysis, food quality control, and biological research. Traditional methods for determining tryptophan, such as high-performance liquid chromatography,^8,9^ capillary electrophoresis,^10^ fluorescence spectroscopy,^11,12^ and colorimetric methods,^13,14^ are highly accurate but usually require complicated sample preparation, costly equipment, lengthy analysis, and skilled operators. These requirements restrict their routine use in rapid and on-site analyses. Conversely, electrochemical sensing techniques have received growing interest because of their simplicity, low cost, high sensitivity, fast response, and potential for miniaturization and portability.^15–17^ Nonetheless, electrochemical oxidation of tryptophan at bare electrodes is usually characterized by a slow electron-transfer rate, low sensitivity, and electrode deactivation; therefore, there is a need to develop improved electrode materials with electrocatalytic activity.^18–20^

CNTs have been extensively studied as electrode modifiers due to their high electrical conductivity, large surface area, chemical stability, and good electron-transfer capability.^21–23^ However, pure CNTs are usually limited in intrinsic electrocatalytic activity toward tryptophan oxidation and mainly serve as conductive supports. To overcome this limitation, the incorporation of catalytically active metal nanoparticles onto CNTs has emerged as an effective strategy for improving the electrochemical performance.^24–26^ Specifically, bimetallic catalysts have often been reported to show enhanced activity compared with their monometallic counterparts due to electronic and geometrical interactions, improved charge-transfer kinetics, and optimized adsorption behavior.^27–29^ Noble metals, such as palladium (Pd), are known for their excellent catalytic activity and hydrogen adsorption ability,^30^ while ruthenium (Ru) possesses favorable redox properties and strong electronic interactions with carbon-based supports.^31,32^ In a bimetallic system, Ru and Pd can form unique electronic structures and strong metal–metal and metal–support interactions, which may lead to improved electrocatalytic activity. Although these advantages exist, studies on Ru–Pd bimetallic catalysts supported on CNTs for electrochemical tryptophan sensing remain limited, and a comprehensive investigation of the structure–property-performance relationship is still needed. Besides experimental studies, DFT computations are now an effective means of explaining the electronic basis of improved electrochemical activity. DFT analysis can provide valuable information about frontier molecular orbitals, energy gaps, charge distribution, and electron localization and therefore can establish a fundamental relationship between material structure and sensing performance. Nevertheless, few studies have combined experimental and theoretical approaches to investigate Ru–Pd/CNT-based electrochemical sensors for tryptophan detection.

In this work, we report the design and fabrication of a highly sensitive electrochemical sensor for tryptophan detection based on a bimetallic Ru–Pd/CNT. The Ru–Pd/CNT nanocomposite was synthesized via a facile NaBH4 reduction method and comprehensively characterized to elucidate its structural, morphological, surface, and redox properties. The catalytic material was employed to modify a GCE, and its electrochemical sensing performance toward tryptophan was systematically evaluated using CV, DPV, and EIS. Furthermore, comparative DFT calculations were conducted for CNT, Pd/CNT, Ru/CNT, and Ru–Pd/CNT to clarify the electronic structure, charge-transfer characteristics, and interfacial electronic effects induced by monometallic and bimetallic metal decoration. The present study provides a clear experimental and theoretical framework for the development of high-performance bimetallic CNT-based electrochemical sensors and highlights the potential of Ru–Pd/CNT systems for sensitive and selective tryptophan determination in biological and analytical applications.

## 2 Experimental Section

### 2.1 Materials and Chemicals

Ruthenium­(III) chloride trihydrate (RuCl_3_.3H_2_O, 99.98%), potassium tetrachloropalladate­(II) (K_2_PdCl_4_, 98%), and sodium borohydride (NaBH_4_, 98%) were purchased from Merck and used as received without further purification. Multiwalled carbon nanotubes (MWCNTs; Nanografi Nanotechnology, product code: NG01MW0314, purity: 92%, outside diameter: 8–10 nm) were used as the carbon support material for catalyst preparation. For consistency with the terminology used throughout the paper, these MWCNTs are referred to as CNTs. The CNTs were used as received without further purification. Nafion solution (D2021) was supplied by The Chemours Company and used as received. Phosphate-buffered saline (PBS, 0.1 M) solutions with the desired pH values were freshly prepared by using analytical-grade reagents and deionized water obtained from a Milli-Q water purification system. All other chemicals employed in this study were of analytical grade and purchased from commercial suppliers.

### 2.2 Synthesis of the Ru–Pd/CNT Catalyst

The Ru–Pd/CNT catalyst was synthesized via a chemical reduction method using NaBH_4_ as the reducing agent. In a typical synthesis, 64.7 mg of RuCl_3_.3H_2_O and 80.8 mg of K_2_PdCl_4_, corresponding to an equimolar ratio (1:1), were dissolved in deionized water and sonicated until a homogeneous solution was obtained. Subsequently, 500 mg of CNTs were added to the metal precursor solution, and the resulting suspension was subjected to combined ultrasonic treatment and magnetic stirring for approximately 2 h to ensure uniform dispersion of the metal species on the CNT surface. Then, 561.0 mg of NaBH_4_, which was equivalent to 30 mol equiv of NaBH_4_ in the total mass of metal precursors (Ru + Pd), was added to the mixture with continuous stirring. The reduction process was allowed to continue for an additional 1 h. The product obtained after the filtration was further washed in deionized water to take out the remaining ions and dried at 85 °C over a period of 12 h to get the Ru–Pd/CNT catalyst.

### 2.3 Characterization of the Ru–Pd/CNT Catalyst

The structural, morphological, compositional, surface, and thermal properties of the Ru–Pd/CNT catalyst were examined using several analytical techniques. Powder X-ray diffraction (XRD) patterns were recorded using a Panalytical Empyrean diffractometer with Cu Kα radiation (λ = 1.5406°A). The diffraction patterns were collected over a 2θ range of 10–90° with a step size of 0.02° and a scanning rate of 2° min^–1^. The crystallite size of the Ru–Pd nanoparticles was estimated from the XRD peak broadening using the Scherrer equation

D=Kλ/β⁡cos⁡θ

where *D* is the crystallite size, *K* is the shape factor, taken as 0.9, λ is the X-ray wavelength, β is the full width at half-maximum (FWHM) in radians, and θ is the Bragg angle.

Scanning electron microscopy combined with energy-dispersive X-ray spectroscopy (SEM-EDX) was used to investigate the surface morphology and elemental composition of the catalyst. SEM images were obtained at an accelerating voltage of 10 kV, a working distance of approximately 10–12 mm, and magnifications ranging from 20,000× to 50,000× to examine the morphology of the CNT support and the distribution of metal-containing features. EDX analysis was performed at an accelerating voltage of 15 kV, and elemental mapping was used to determine the spatial distribution of carbon (C), ruthenium (Ru), and palladium (Pd) in the catalyst. Inductively coupled plasma mass spectrometry (ICP-MS) was used to determine the elemental composition and metal loading of the samples after suitable acid digestion using a Thermo iCAP RQ ICP-MS device. Transmission electron microscopy (TEM) analysis was performed using a JEOL JEM-1220 microscope operated at an accelerating voltage of 100 kV to further examine the nanoscale morphology and dispersion of Ru–Pd nanoparticles on the CNT support. X-ray photoelectron spectroscopy (XPS) was performed using a SPECS Flex system with a monochromatic Al Kα X-ray source (*h*ν = 1486.6 eV) to determine the surface elemental composition and oxidation states of the Ru–Pd/CNT catalyst. Survey spectra were recorded over a binding energy range of 0–1400 eV, while high-resolution spectra were collected for C 1s, Ru 3p, Pd 3d, and O 1s regions. The binding energies were normalized to the C 1s peak at 284.8 eV. The reducibility, oxidizability, and surface acidity of the Ru–Pd/CNT catalyst were investigated by temperature-programmed reduction (H_2_-TPR), temperature-programmed oxidation (O_2_-TPO), and temperature-programmed desorption (NH_3_-TPD) analyses using a ChemiSorb 2720 instrument, respectively. Approximately 100 mg of catalyst was used for each measurement. H_2_-TPR experiments were performed by heating the sample from room temperature to 800 °C at a rate of 10 °C min^–1^ under a 10 vol % H_2_/Ar gas flow. O_2_-TPO measurements were carried out under a 10 vol % O_2_/He atmosphere using the same temperature program. NH_3_-TPD analysis involved NH_3_ adsorption at 100 °C, followed by desorption from 100 to 800 °C at a heating rate of 10 °C min^–1^ under a helium flow.

### 2.4 Electrochemical Measurements

Electrochemical measurements were performed using a three-electrode configuration consisting of a GCE (3 mm diameter) as the working electrode, a platinum wire as the counter electrode, and an Ag/AgCl electrode as the reference electrode. All experiments were conducted in 0.1 M phosphate-buffered saline (PBS) using a CHI 660E electrochemical workstation at room temperature. Prior to modification, the GCE surface was polished sequentially with alumina slurries of decreasing particle size, thoroughly rinsed with deionized water, and dried under ambient conditions. For electrode modification, 5 mg of Ru–Pd/CNT catalyst was ultrasonically dispersed in 0.5 mL of Nafion solution (5 wt % in 1-propanol) to obtain a homogeneous suspension. Subsequently, 3 μL of the suspension was drop-cast onto the cleaned GCE surface and allowed to dry at room temperature, yielding the Ru–Pd/CNT@GCE. For comparison and electroactive surface area (ECSA) evaluation, CNT@GCE, Pd/CNT@GCE, and Ru/CNT@GCE control electrodes were prepared using the same drop-casting procedure by replacing Ru–Pd/CNT with CNT, Pd/CNT, or Ru/CNT, respectively. Bare GCE was also evaluated under the same conditions as a reference electrode. CV measurements were carried out to investigate the electrochemical behavior of the Ru–Pd/CNT@GCE sensor. CV was employed to compare the electrooxidation responses of different amino acids, to compare the tryptophan oxidation responses of bare GCE, CNT@GCE, Pd/CNT@GCE, Ru/CNT@GCE, and Ru–Pd/CNT@GCE, to investigate the concentration-dependent electrooxidation behavior of tryptophan, and to examine the influence of scan rate on the electrochemical response of the Ru–Pd/CNT@GCE sensor. Unless otherwise stated, all CV measurements were carried out in 0.1 M PBS at a scan rate of 100 mV/s. Quantitative determination of tryptophan was performed using DPV. DPV measurements were conducted in 0.1 M PBS (pH 7.0) over a tryptophan concentration range of 1–1200 μM using a pulse amplitude of 50 mV, pulse width of 50 ms, sampling width of 16.7 ms, pulse period of 200 ms, and potential increment of 4 mV. Interference studies were performed by DPV using tryptophan (Trp) in the absence and presence of ascorbic acid (AA), uric acid (UA), glucose (Glu), and their mixed interferent solution in 0.1 M PBS (pH 7.0). The same DPV parameters used for tryptophan determination were applied, and the relative response was calculated from the Trp oxidation peak current. Calibration curves were constructed from the peak current responses obtained at different tryptophan concentrations. The sensitivity and LOD were calculated using the following equations

sensitivity=S

LOD=3.3σ/S

where *S* represents the slope of the calibration curve and σ is the standard deviation of the intercept obtained from the calibration curve.

EIS measurements were conducted to examine the interfacial electrochemical properties of the Ru–Pd/CNT@GCE sensor. EIS spectra were recorded in 0.1 M PBS (pH 7.0) containing 5 mM tryptophan by applying an AC perturbation of 5 mV over a frequency range of 0.1 Hz to 100 kHz at an applied potential of 0.8 V.

The ECSA of bare GCE, CNT@GCE, Pd/CNT@GCE, Ru/CNT@GCE, and Ru–Pd/CNT@GCE was evaluated by CV in 5 mM K_3_[Fe­(CN)_6_] containing 0.1 M KCl at scan rates of 20, 40, 60, 80, and 100 mV/s. The anodic peak current (*I*_pa_) was extracted from each voltammogram and plotted against the square root of the scan rate (ν^1/2^). The ECSA values were then calculated using the Randles–Sevcik equation.

Chronoamperometric stability was evaluated at an applied potential of 0.8 V in 0.1 M PBS containing 5 mM tryptophan for 2600 s to assess the short-term operational stability of the Ru–Pd/CNT@GCE sensor.

### 2.5 DFT Calculations

DFT calculations were carried out to investigate the structural and electronic properties of pristine CNT, monometallic Pd/CNT, monometallic Ru/CNT, and bimetallic Ru–Pd/CNT systems and to provide theoretical support for the experimentally observed electrochemical sensing behavior. The present DFT results are therefore intended as complementary support for the experimentally observed performance of the specific CNT-based catalyst systems studied here, rather than as a basis for generalizing the behavior of all metal-decorated CNT materials. All quantum chemical calculations were performed using the Gaussian 16 software package,^33,34^ and molecular structures as well as orbital visualizations were generated with GaussView 6.0.^35^ Finite cluster models were constructed to represent the local surface structure of CNT. Since the experimentally used CNT support consists of commercial multiwalled carbon nanotubes (MWCNTs), the finite cluster model was designed as a simplified CNT-wall segment representing the local graphitic surface environment of the MWCNTs. Therefore, the model was used to describe local Pd/CNT, Ru/CNT, and Ru–Pd/CNT electronic interactions and charge redistribution, rather than to reproduce the complete multiwalled morphology, diameter distribution, or chirality-dependent electronic properties of the commercial CNT material. For the Pd/CNT, Ru/CNT, and Ru–Pd/CNT systems, Pd, Ru, or both Ru and Pd atoms were initially placed on the CNT surface to model the corresponding metal–support interactions and were fully relaxed during geometry optimization. All geometry optimizations were conducted without symmetry constraints using the B3LYP hybrid exchange–correlation functional, selected for its reliable balance between accuracy and computational efficiency in carbon-based systems. The LANL2DZ effective core potential basis set was employed for all atoms (C, H, Ru, and Pd) to ensure methodological consistency among the pristine CNT, Pd/CNT, Ru/CNT, and Ru–Pd/CNT models and to provide a uniform description of electronic interactions. Vibrational frequency calculations were performed following geometry optimization to confirm that all optimized structures correspond to true minima on the potential energy surface, as evidenced by the absence of imaginary frequencies.^36–38^ Frontier molecular orbital energies (*E*_HOMO_ and *E*_LUMO_) were obtained from the optimized geometries, and global reactivity descriptors, including the energy gap (Δ*E* = *E*_LUMO_ – *E*_HOMO_), chemical potential (μ = [*E*_HOMO_ + *E*_LUMO_]/2), chemical hardness (η = [*E*_LUMO_ – *E*_HOMO_]/2), and electrophilicity index (ω = μ^2^/2η), were calculated to assess the electronic reactivity of the systems.^39–41^ MEP maps were generated to visualize the charge distribution and identify electrophilic and nucleophilic regions relevant to analyte–surface interactions.^42,43^ In addition, ELF and LOL analyses were performed to explore electron localization–delocalization behavior and metal–support electronic interactions.^44,45^ The Multiwfn program was used to perform all of the MEP, ELF, and LOL analyses based on the wave function files generated by Gaussian calculations.

## 3 Results and Discussion

### 3.1 Characterization of the Ru–Pd/CNT Catalyst

A systematic physicochemical characterization of the prepared Ru–Pd/CNT catalyst was carried out to elucidate its structural, morphological, surface, and redox properties. A combination of XRD, SEM-EDX, XPS, and temperature-programmed techniques (H_2_-TPR, O_2_-TPO, and NH_3_-TPD) was employed to obtain a comprehensive understanding of the phase composition, metal dispersion, surface chemical states, and metal–support interactions of the catalyst.

XRD was used to determine the crystal structure and phase composition of the synthesized Ru–Pd/CNT catalyst, and the related diffraction pattern is presented in Figure 1. The unit diffraction peak at 2θ = 25.7° is attributed to the (002) of graphitic carbon, which validates the typical crystalline structure of the CNT support (JCPDS card no. 96–101–1061).^46^ This finding shows that the CNT structure is structurally stable once the bimetallic constituents have been deposited on it. In addition to the CNT-related reflection, several diffraction peaks corresponding to Pd were also observed. The peaks appearing at 2θ = 40.0°, 45.6°, 67.0°, and 81.5° correspond to the (111), (200), (220), and (311) planes of face-centered cubic (fcc) Pd, respectively (JCPDS card No. 46–1043).^47^ The existence of these reflections proves the effective creation of crystalline Pd nanoparticles that were suspended on the surface of CNT. Furthermore, the instances of diffraction at 2θ = 43.0° and 2θ = 78.2° are assigned to the presence of ruthenium nanoparticles and can be referred to the (101) and (103) planes, respectively, in accordance with the reported diffraction pattern of metallic Ru (JCPDS card no. 06–0663).^48^ The simultaneous detection of characteristic diffraction peaks for both Ru and Pd demonstrates the effective incorporation of the bimetallic species within the CNT-supported catalyst. The absence of additional impurity-related diffraction peaks suggests that the Ru–Pd/CNT catalyst was synthesized with high phase purity. Furthermore, the relatively broad and low-intensity nature of the metal-related peaks implies a nanoscale crystallite size and good dispersion of Ru and Pd particles on the CNT support. Such structural features are beneficial for electrochemical applications, as they enhance the availability of active sites and promote efficient electron transport through the conductive CNT network.

**Figure 1:**
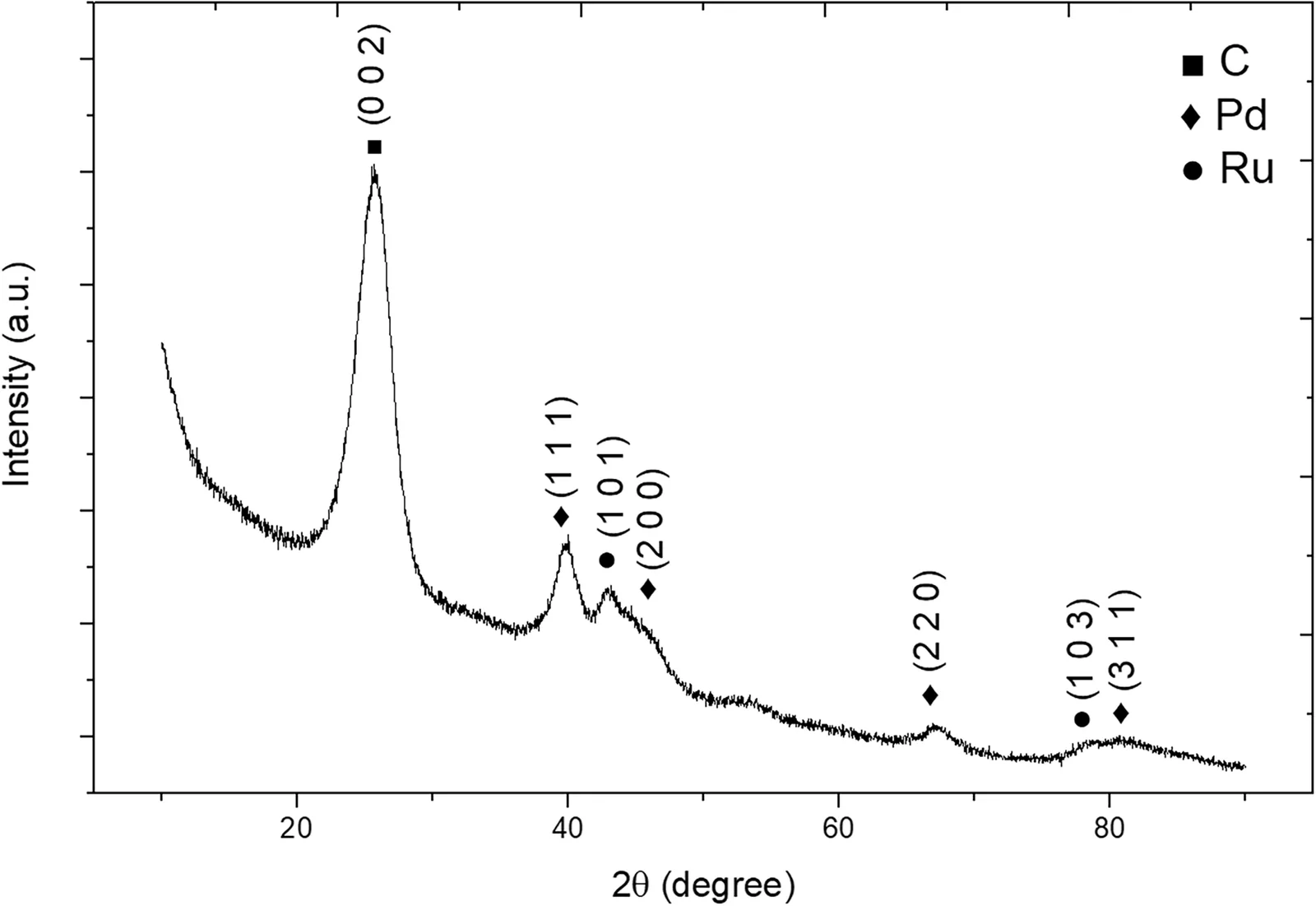
XRD pattern of the Ru–Pd/CNT catalyst.

To further evaluate the nanoscale crystalline nature of the deposited metal species, the crystallite size was calculated from the XRD peak broadening using the Scherrer equation, and the results are summarized in Table 1. The Pd(111), Ru(101), and Pd(220) reflections were selected for the calculation because they were relatively clear and distinguishable. The calculated crystallite sizes were approximately 5.52, 7.13, and 5.65 nm, respectively, giving an average crystallite size of ∼6.4 nm. These values confirm that the Ru–Pd species are present as nanoscale crystallites. Therefore, the broad and low-intensity metal-related diffraction peaks are consistent with the formation of small Ru–Pd crystallites on the CNT support.

**Table 1:** Crystallite Size Estimation of Ru–Pd/CNT from XRD Peak Broadening Using the Scherrer Equation

reflection	2θ position	FWHM	crystallite size
Pd (111)	∼39.8–40.0°	∼1.5°	5.52 nm
Ru (101)	∼43.0°	∼1.2°	7.13 nm
Pd (220)	∼67.0°	∼1.6°	5.65 nm

Figure 2 presents SEM images of the Ru–Pd/CNT catalyst recorded at magnifications of 20,000× (Figure 2a) and 50,000× (Figure 2b), providing information on its surface morphology and structural characteristics. When the magnification is smaller (Figure 2a), a very interconnected and entangled network of CNT is observed, creating a porous three-dimensional structure.^49,50^ This structure is typical of CNT-based materials and offers a notable benefit to electrochemical applications since it provides a high accessible surface area and facilitates mass and electron transport. The increased magnification SEM image (Figure 2b) indicates that the surfaces of CNTs are decorated with metal-containing features attributed to Ru–Pd species. Although SEM confirms the presence of metal-containing features on the CNT surface, its resolution is not sufficient to clearly distinguish individual nanoparticles from aggregates of smaller crystallites. Therefore, the larger features observed in SEM may correspond to agglomerates composed of nanoscale Ru–Pd crystallites, as supported by the Scherrer crystallite size estimation. The preservation of the CNT structure despite the addition of the metal also implies that the synthesis process does not negatively affect the carbon skeleton, which is essential for maintaining the high electrical conductivity and mechanical stability of the structure.

**Figure 2:**
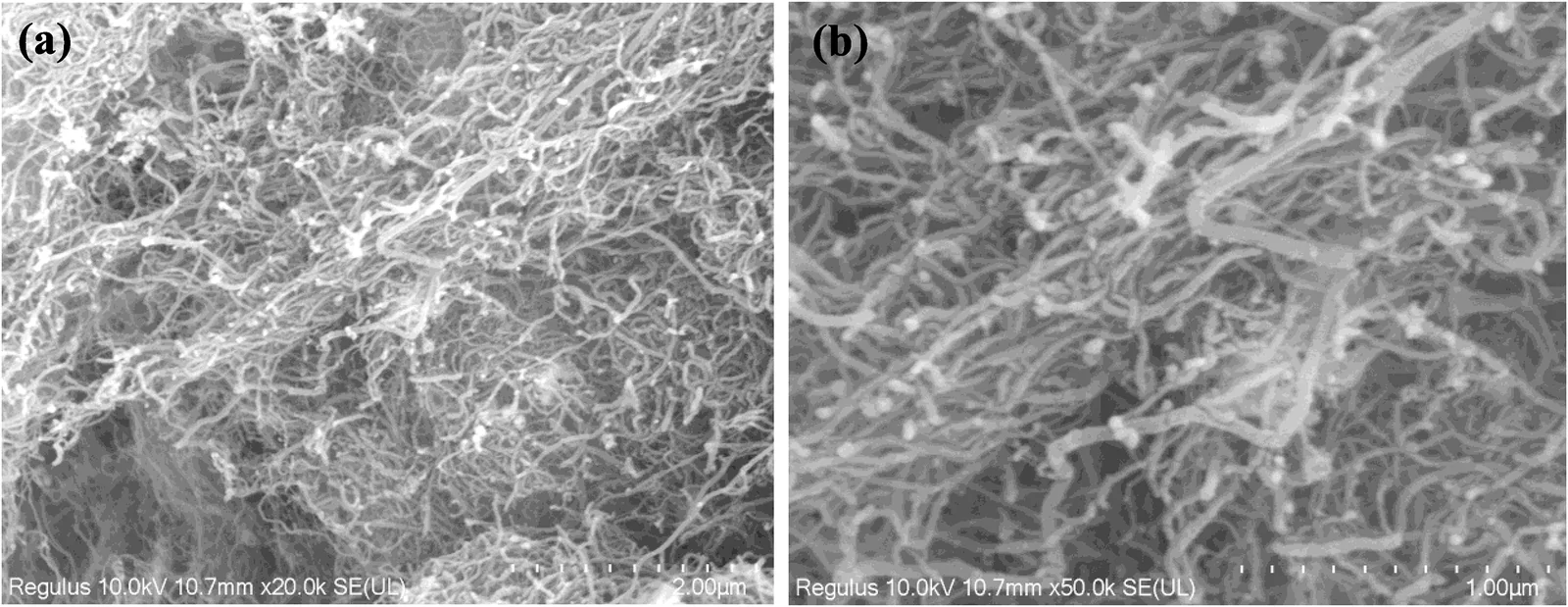
SEM images of the Ru–Pd/CNT catalyst recorded at different magnifications: (a) 20,000× and (b) 50,000×.

Figure 3 displays the elemental mapping analysis of the elemental composition and spatial distribution of the Ru–Pd/CNT catalyst based on the EDX spectrum, which gives an understanding of its elemental composition and distribution. Figure 3a indicates a strong signal of carbon (C) at the EDX spectrum that is attributed to the CNT support, together with the characteristic peaks of oxygen (O), ruthenium (Ru), and palladium (Pd). The peaks are observed at approximately 0.28 keV (C Kα), 0.52 keV (O Kα), 2.56 keV (Ru Lα), and 2.84–3.00 keV (Pd L lines). In addition, weak signals at approximately 1.49 keV (aluminum (Al) Kα) and 2.12 keV (gold (Au) Mα) are also observed. The O signal is attributed to oxygen-containing surface species, including oxygen functionalities on the CNT support and partially oxidized Ru/Pd species, whereas the Al and Au signals originate from the Al sample holder and the Au coating used during SEM-EDX sample preparation, respectively. Therefore, these minor Al and Au signals are not related to the intrinsic composition of the Ru–Pd/CNT catalyst. The overall CNT network structure and the localization of the bimetallic components within the region being studied are given in the composite elemental map in Figure 3b. Because Figure 3b represents a composite elemental overlay of C, O, Ru, and Pd, the visual appearance of the carbon signal is influenced by the overlap of the other elemental channels as well as the contrast and color scaling used in the composite rendering. In contrast, Figure 3c shows only the carbon map; therefore, the CNT framework appears more continuous and pronounced. Thus, the apparent difference between Figure 3b[Fig fig3],c is a visualization effect rather than a real difference in carbon distribution. The carbon elemental map (Figure 3c) indicates a uniform and continuous distribution, which indicates that the CNT framework is still intact and uniformly spread after metal loading. This type of continuous carbon network is important in the process of facilitating the efficient movement of electrons and electrical conductivity. The O elemental map (Figure 3d) shows a homogeneous distribution over the CNT network, supporting the presence of surface oxygen-containing species throughout the catalyst structure. Both metal species are uniformly spread on the CNT surface, as the Ru (Figure 3e) and Pd (Figure 3f) elemental maps do not show any signs of localized enrichment or agglomeration of particles. The strong spatial overlap between the Ru and Pd distributions indicates successful codeposition of the bimetallic components and suggests intimate contact between the two metals at the nanoscale, which is favorable for interfacial electronic interaction and improved catalytic behavior. The EDX spectrum and elemental mapping results in Figure 3 confirm the successful fabrication of a uniformly distributed Ru–Pd bimetallic catalyst supported on CNTs, forming a robust structural basis for the enhanced electrochemical performance discussed in subsequent sections.

**Figure 3:**
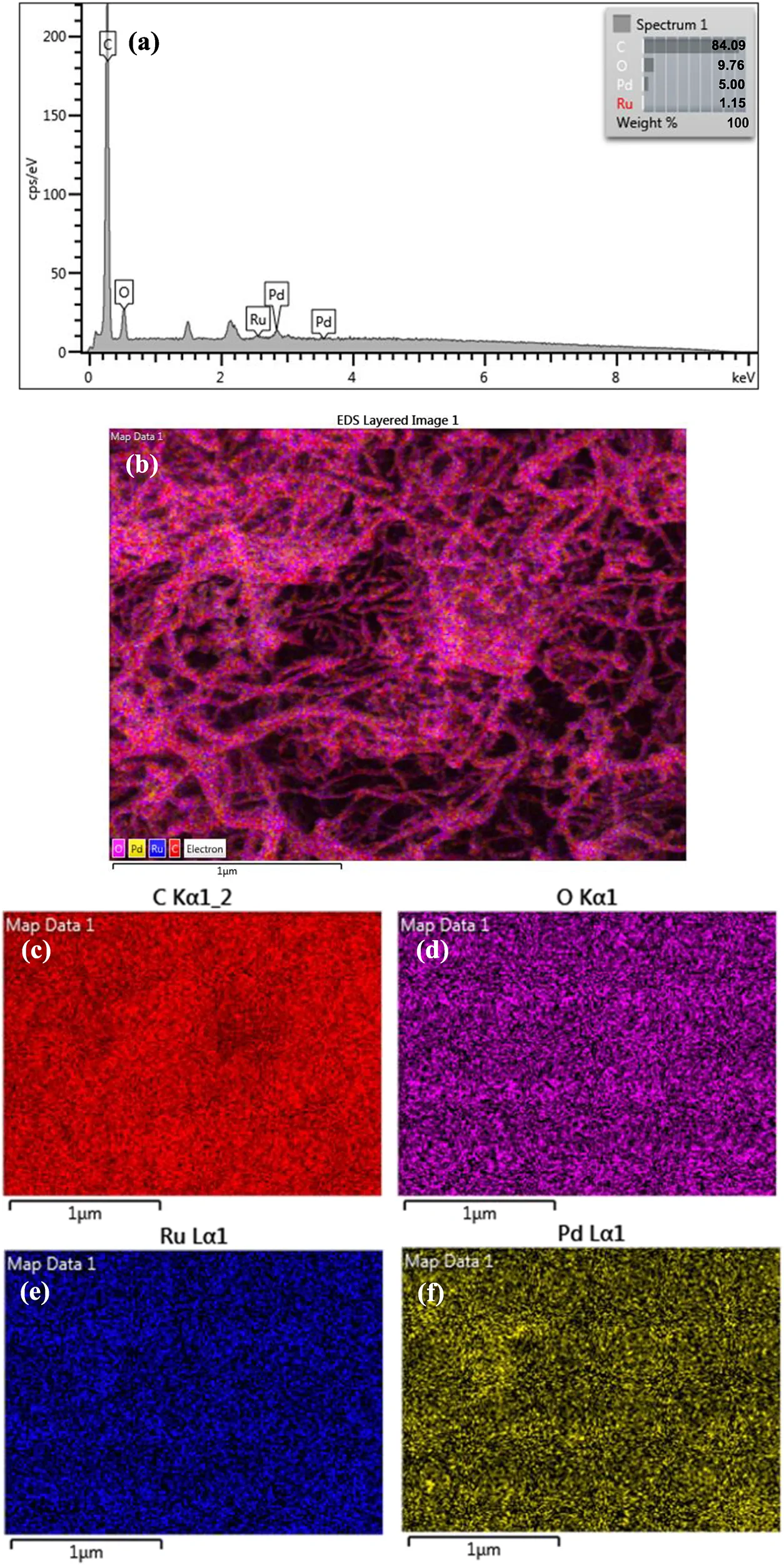
EDX spectrum and elemental mapping analysis of the Ru–Pd/CNT catalyst: (a) EDX spectrum, (b) composite elemental map, and elemental distribution maps of (c) C, (d) O, (e) Ru, and (f) Pd.

As EDX provides surface-sensitive and semiquantitative information, it was primarily employed to confirm elemental presence and spatial homogeneity. Accurate determination of the metal composition was therefore carried out by inductively coupled plasma mass spectrometry (ICP-MS), which revealed a Ru:Pd atomic ratio of 53.2% Ru and 46.8% Pd. This value is in good agreement with the targeted equimolar (1:1) composition, confirming the effectiveness of the synthesis strategy for achieving controlled bimetallic loading. Since SEM-EDX provides local and surface-sensitive semiquantitative information, whereas ICP-MS determines the bulk metal composition after acid digestion, slight differences between the Ru/Pd ratios obtained from these two techniques are reasonable.

TEM analysis was further performed to directly examine the nanoscale morphology and dispersion of Ru–Pd nanoparticles on the CNT support. As shown in Figure 4, the Ru–Pd/CNT catalyst exhibits an entangled CNT network decorated with small dark nanoparticles. These dark contrast features can be attributed to Ru–Pd species because of the higher electron density of Ru and Pd compared with that of the carbon support. At lower magnification (Figure 4a), the interconnected CNT network is clearly observed, while the higher-magnification TEM images (Figure 4b–d) reveal that small Ru–Pd nanoparticles are distributed on the CNT surface. Some localized aggregation can also be observed, indicating that the larger features seen in SEM may correspond to agglomerates composed of smaller nanoscale crystallites rather than individual single nanoparticles. This observation is consistent with the XRD-based Scherrer analysis, which indicated nanoscale Ru–Pd crystallites with an average crystallite size of approximately 6.4 nm. Therefore, the combined XRD, SEM, EDX/ICP-MS, and TEM results confirm the successful formation of a Ru–Pd/CNT nanocomposite with nanoscale metal crystallites distributed on the CNT framework.

**Figure 4:**
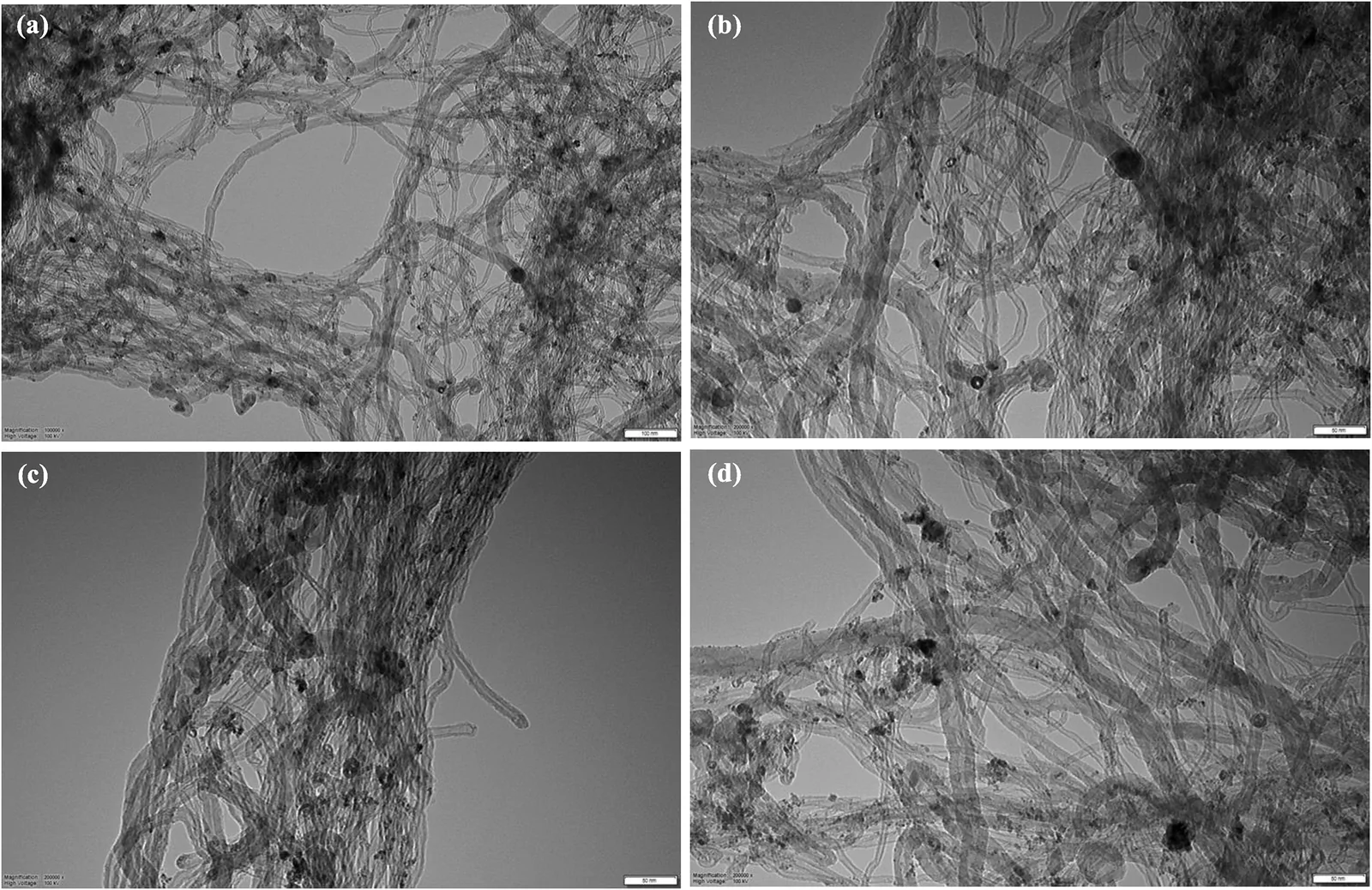
TEM images of the Ru–Pd/CNT catalyst: (a) 100,000×, 100 nm; (b–d) 200,000×, 50 nm.

To further support the TEM analysis, the CNT diameter distribution was estimated from the high-magnification TEM image in Figure 4b by using ImageJ software. The diameters of individual CNTs were measured perpendicular to the tube axis, and the corresponding size distribution histogram is shown in Figure 5. The average CNT diameter was calculated to be approximately 9.38 nm, confirming the nanoscale tubular morphology of the CNT support and its consistency with the expected diameter range of the commercial CNT material.

**Figure 5:**
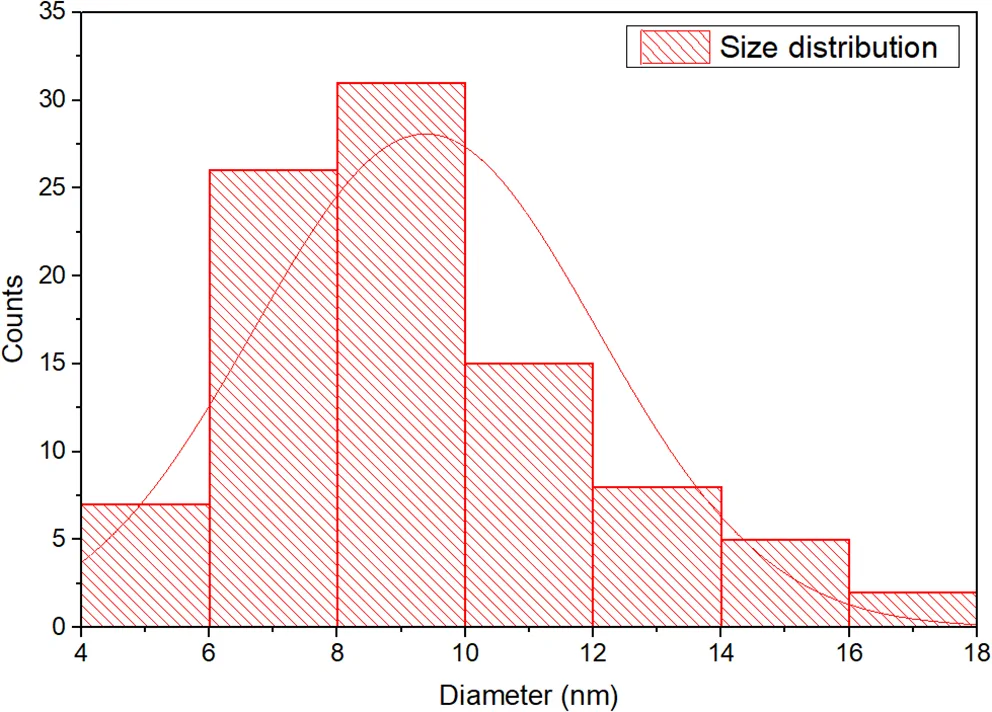
CNT diameter distribution histogram obtained from the TEM image in Figure 4b using ImageJ software.

XPS (Figure 6) was employed to investigate the surface chemical composition and oxidation states of the Ru–Pd/CNT catalyst. Figure 6a on the XPS survey spectrum proves the existence of C, O, Ru, and Pd, which confirms the successful loading of the bimetallic species on the CNT support. According to the high-resolution C 1s spectrum (Figure 6b), the predominant peak is at 284.4 eV, which is considered to be on the *sp* ^2^-hybridized carbon (C=C) in the graphitic structure of CNTs.^51^ The presence of a weaker feature at high binding energy, which is attributed to π–π* shakeup transitions, demonstrates the aromatic character of the CNT backbone and ensures the maintenance of the graphitic carbon backbone after the synthesis of the catalyst. Two typical spin–orbit doublets are visible in the Pd 3d core-level spectrum (Figure 6c). The two peaks at 335.6 eV (Pd 3d_5/2_) and 341.3 eV (Pd 3d_3/2_) are attributed to metallic palladium (Pd^0^), and the other two peaks at 337.1 and 343.8 eV are due to the oxidized palladium species (PdO).^52^ The coexistence of Pd^0^ and PdO indicates partial surface oxidation of Pd nanoparticles, as frequently observed in supported Pd-based catalysts, and may contribute to the increased electrochemical activity through metal/oxide interfacial interactions. The spectrum of the Ru (Ru 3p/Figure 6d) also indicates the mixed oxidation states of Ru. The metallic Ru^0^ species are identified as the binding energies at 462.9 eV (Ru 3p_3/2_) and 487.7 eV (Ru 3p_1/2_), but the binding energies are 467.3 and 487.0 eV, attributed to the oxidized species, Ru^4+^.^53^ Ru^0^ and Ru^4+^ were present; hence, ruthenium was partially oxidized on the catalyst surface and could facilitate the transfer of electrons and enhance catalytic activity. These XPS findings, which are consistent with the XRD, SEM-EDX, elemental mapping, and ICP-MS data, establish that the uptake of the bimetallic Ru–Pd/CNT catalyst with the desired composition and surface chemical states was achieved successfully. The presence of metallic and oxidized Ru and Pd species on the support of CNTs is likely to be among the primary factors that explain the improvement in the electrochemical behavior of the Ru–Pd/CNT-based sensor.

**Figure 6:**
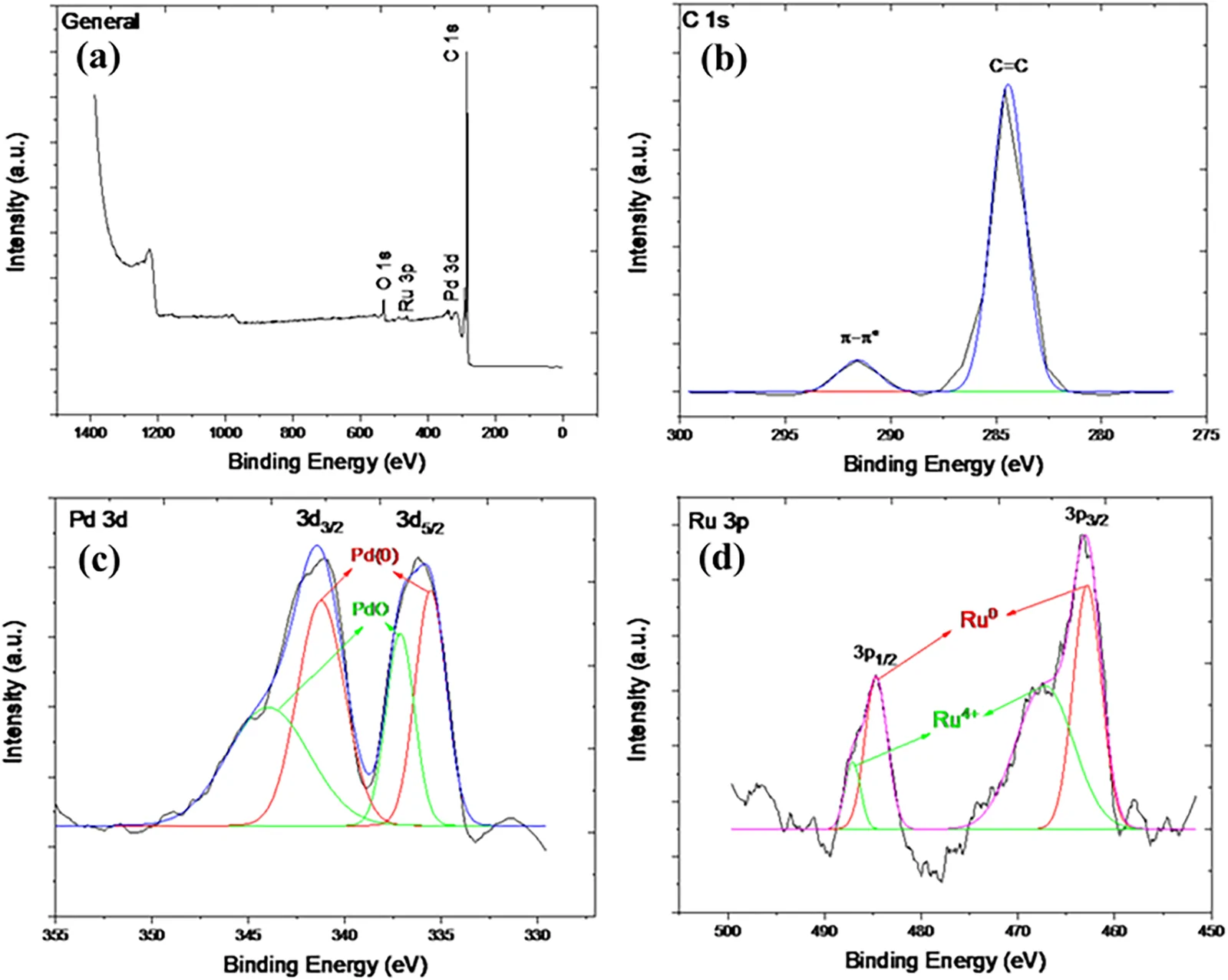
XPS analysis of the Ru–Pd/CNT catalyst: (a) survey spectrum, (b) high-resolution C 1s spectrum, (c) Pd 3d core-level spectrum, and (d) Ru 3p core-level spectrum.

The redox behavior and surface acidity of the Ru–Pd/CNT catalyst were examined with the help of H_2_ temperature-programmed reduction (H_2_-TPR), O_2_ temperature-programmed oxidation (O_2_-TPO), and NH_3_ temperature-programmed desorption (NH_3_-TPD), and presented in Figure 7a–c, respectively. H_2_-TPR profile of the Ru–Pd/CNT catalyst (Figure 7a) shows that the catalyst has several reduction characteristics that are linked to the bimetallic constituents and the interaction between these constituents and the CNT support. It has a strong negative peak maximum at around 80 °C, which is attributed to the hydrogen desorption by the decomposition of palladium hydride (PdH_X_), indicating the strong ability of Pd species to absorb hydrogen.^54,55^ Besides, two hydrogen consumption peaks recorded at approximately 118 and 205 °C are attributed to the stepwise reduction of ruthenium species between Ru^4+^ and Ru^0^, which demonstrates the existence of Ru in other oxidation states. The reduction of Pd^2^ to Pd^0^ is reduced more at a broader reduction peak of about 450 °C. The appearance of multiple reduction events and their temperature shifts suggests metal–metal and metal–support interactions between Ru, Pd, and the CNT matrix, which can influence the catalytic and electrochemical behavior of the material.^56,57^ Figure 7b is the O_2_-TPO profile, which also informs about the oxidation behavior of the catalyst (Ru–Pd/CNT). The slow rate of oxygen consumption at tempering temperatures of 200 to 450 °C is accompanied by the oxidation of the remaining carbonaceous species on the CNT surface. The thermal decomposition of palladium oxide (PdO) is said to be the cause of a separate peak in the oxygen uptake at about 475 °C, whereas the bulk oxidation of metallic ruthenium to RuO_2_ is attributed to a separate peak at about 490 °C.^58,59^ These findings support the existence of oxidation species of metal and point out the variations in oxidation stability of Ru and Pd in the bimetallic system. The surface acidity of the Ru–Pd/CNT catalyst was measured using the NH_3_-TPD profile of the catalyst (Figure 7c). The clear peak of NH_3_ desorption at around 380 °C shows that there are medium-strength acid sites on the catalyst surface.^60^ These acid sites may be correlated to metal–support interfacial areas and partially oxidized metal species, which may lead to an increase in adsorption and electrochemical activity. Taken together, the H_2_-TPR, O_2_-TPO, and NH_3_-TPD findings indicate that the Ru–Pd/CNT catalyst has a complicated redox behavior, robust interactions between its metals and support, and moderate surface acidity. These physicochemical properties are anticipated to be of great importance in supporting the effective transfer of charge and catalytic reactions when used as an electrochemical sensor.

**Figure 7:**
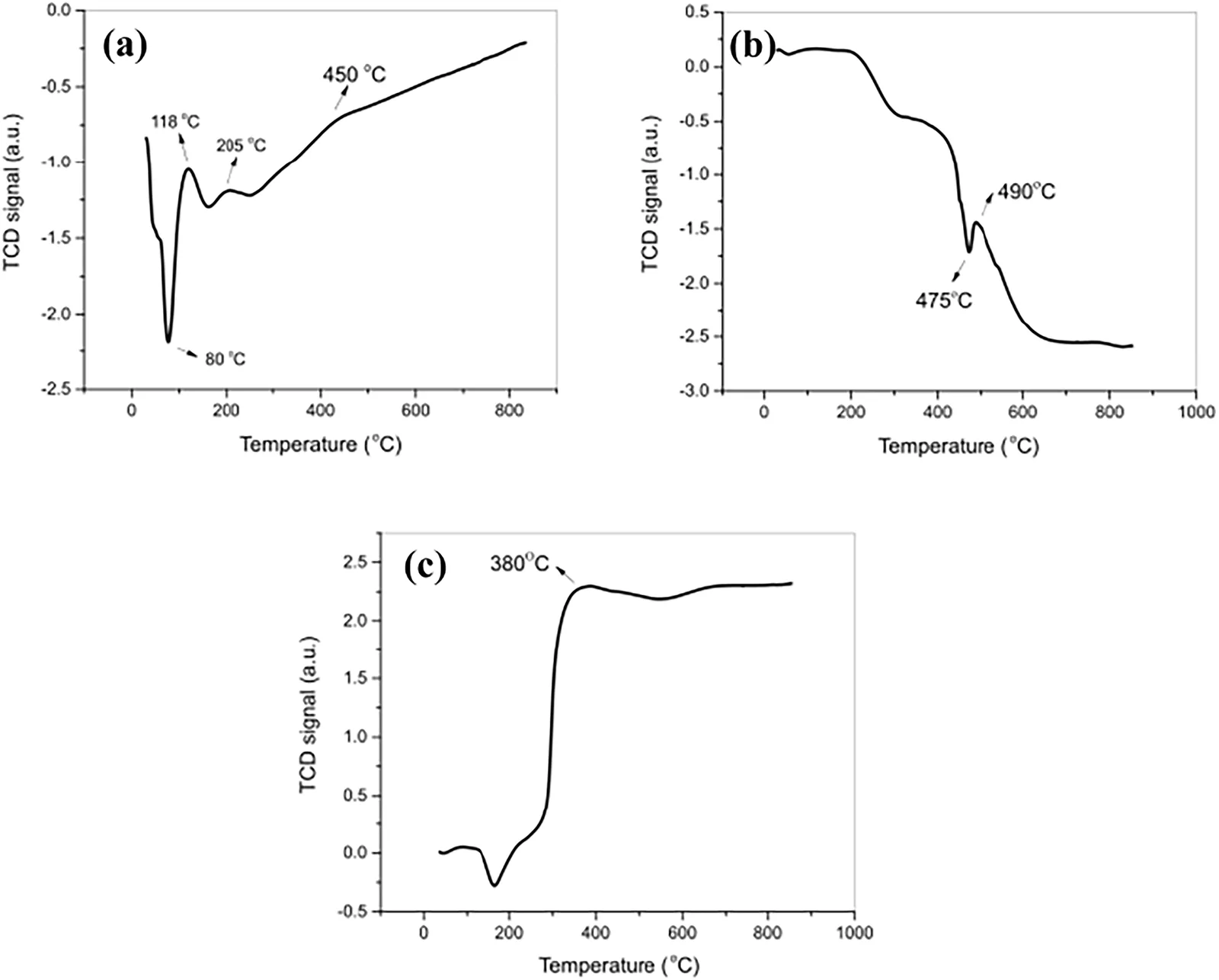
Temperature-programmed analyses of the Ru–Pd/CNT catalyst: (a) H_2_-TPR profile, (b) O_2_-TPO profile, and (c) NH_3_-TPD profile.

### 3.2 Electrochemical Sensing Performance

#### 3.2.1 Comparative Response of Different Electrodes

To evaluate the contribution of the bimetallic Ru–Pd/CNT structure to tryptophan oxidation, the CV responses of bare GCE, CNT@GCE, Pd/CNT@GCE, Ru/CNT@GCE, and Ru–Pd/CNT@GCE were compared under identical experimental conditions. As shown in Figure 8, bare GCE exhibited a weak current response, indicating a limited electrocatalytic activity toward tryptophan oxidation. CNT@GCE showed a slightly improved response, which can be attributed to the conductive nature and surface area of the CNTs’ support. The monometallic Pd/CNT@GCE and Ru/CNT@GCE electrodes displayed higher current responses than those of CNT@GCE, confirming the contribution of metal species to the electrooxidation process. Among all investigated electrodes, Ru–Pd/CNT@GCE exhibited the highest oxidation current density, demonstrating the beneficial effect of the bimetallic Ru–Pd/CNT structure. This enhanced response confirms that the superior electrochemical performance is not only related to the CNT support but also to the combined contributions of Ru and Pd species on the CNT surface. Therefore, Ru–Pd/CNT@GCE was selected for further tryptophan-sensing studies.

**Figure 8:**
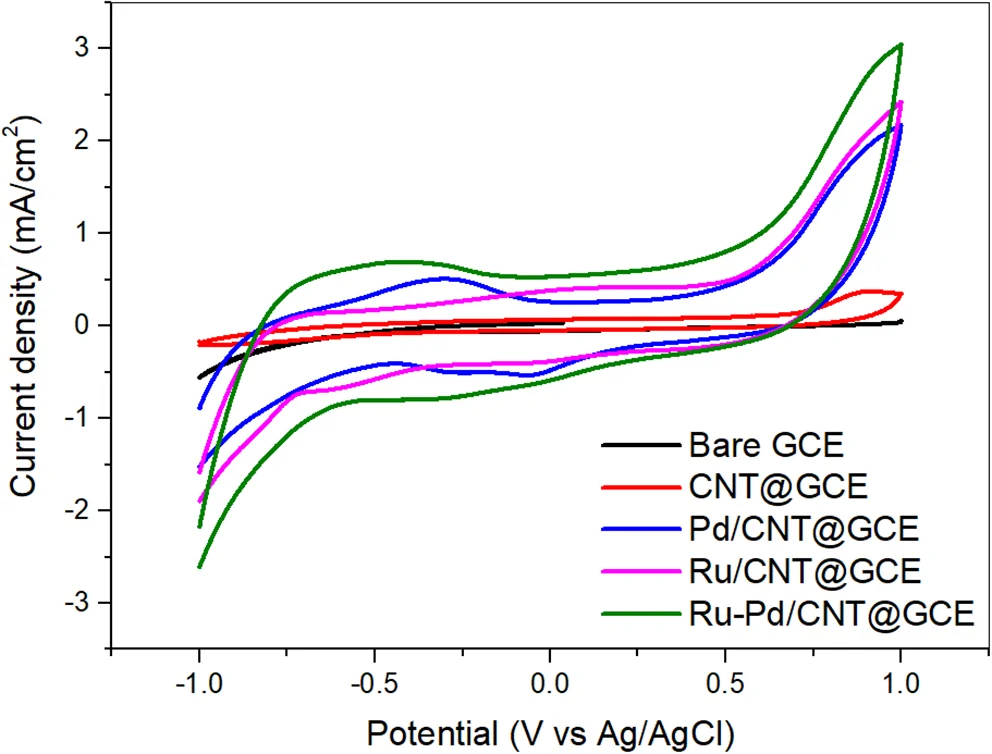
Comparative CV responses of bare GCE, CNT@GCE, Pd/CNT@GCE, Ru/CNT@GCE, and Ru–Pd/CNT@GCE in 0.1 M PBS containing 5 mM tryptophan at a scan rate of 100 mV/s.

#### 3.2.2 Selectivity toward Amino Acids

The electrochemical characteristics of Ru–Pd/CNT@GCE for various amino acids were first determined through CV to determine the selectivity of the sensor. Essential amino acids, histidine (His), isoleucine (Ile), leucine (Leu), lysine (Lys), methionine (Met), phenylalanine (Phe), threonine (Thr), and tryptophan (Trp) were measured using CV at a scan rate of 100 mV/s in 0.1 M phosphate-buffered saline (PBS, pH 7.2), and the response of these amino acids was recorded, as shown in Figure 9. The CV curves show that the Ru–Pd/CNT@GCE sensor exhibits distinct oxidation responses toward the investigated amino acids, with tryptophan giving the highest current response among them. The highest current density of oxidation was recorded on tryptophan at 3.01 mA/cm^2^, and this was by a wide margin higher than the current density responses of His, Ile, Leu, Lys, Met, Phe, and Thr. This significant increase in current suggests that the surface of Ru–Pd/CNT is very electrocatalytically active in the oxidation of tryptophan and reflects the selectivity nature of the sensor. Such an enhancement in the electrochemical behavior toward tryptophan can be explained by the combined contribution of Ru–Pd bimetallic species and the conductive CNT network, which facilitates efficient electron transfer and favorable adsorption of tryptophan molecules on the electrode surface. Conversely, the other amino acids show fairly weak and less pronounced redox reactions under the same conditions, indicating the lower electrochemical activity at the Ru–Pd/CNT interface at the GCE. On the basis of these comparative CV results, tryptophan was selected as the target analyte for further electrochemical investigations. Subsequent studies were therefore focused on evaluating the effect of tryptophan concentration, scan rate dependence, and interference from common biological interferents to comprehensively assess the sensing performance of the Ru–Pd/CNT@GCE sensor.

**Figure 9:**
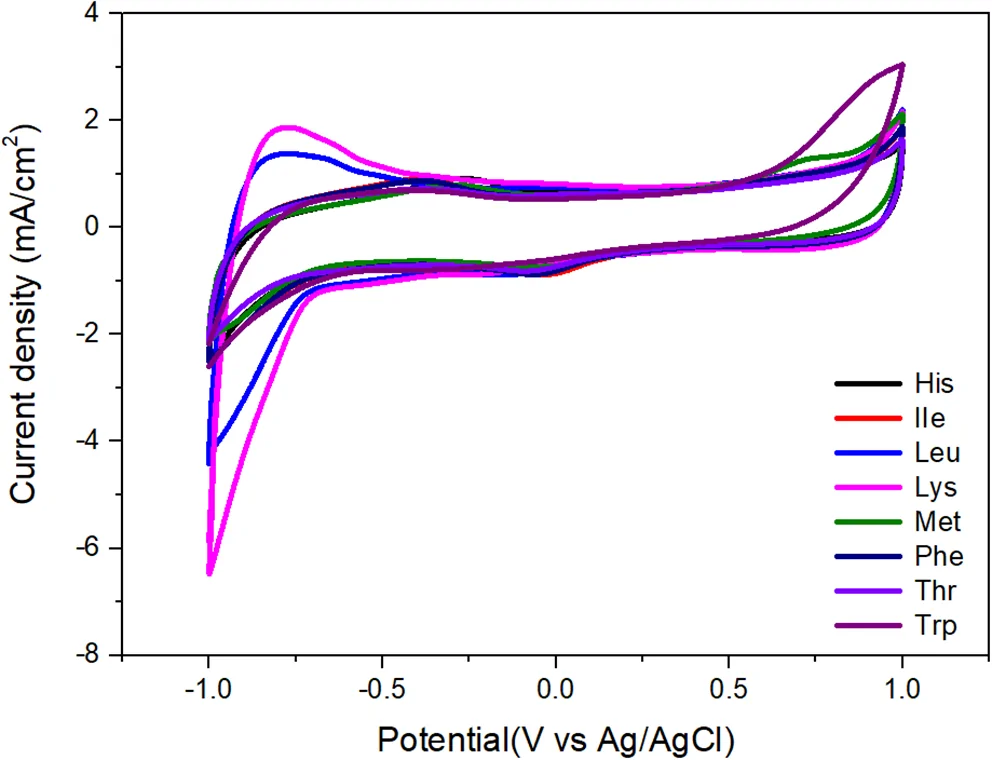
Cyclic voltammograms recorded for different amino acids at the Ru–Pd/CNT@GCE sensor in 0.1 M PBS (pH 7.2) at a scan rate of 100 mV/s.

#### 3.2.3 Interference Study

The anti-interference ability of the Ru–Pd/CNT@GCE sensor was further evaluated using DPV in the presence of common biological interferents, including ascorbic acid (AA), uric acid (UA), glucose (Glu), and their mixed interferent solution. DPV responses were recorded for tryptophan (Trp) alone and in the presence of AA, UA, Glu, and the mixed interferents in 0.1 M PBS (pH 7.0). As shown in Figure 10, the addition of these interferents did not produce a distinct new oxidation peak overlapping with the Trp response region. Although slight variations in the current response were observed, the overall DPV profiles remained comparable to those of Trp alone.

**Figure 10:**
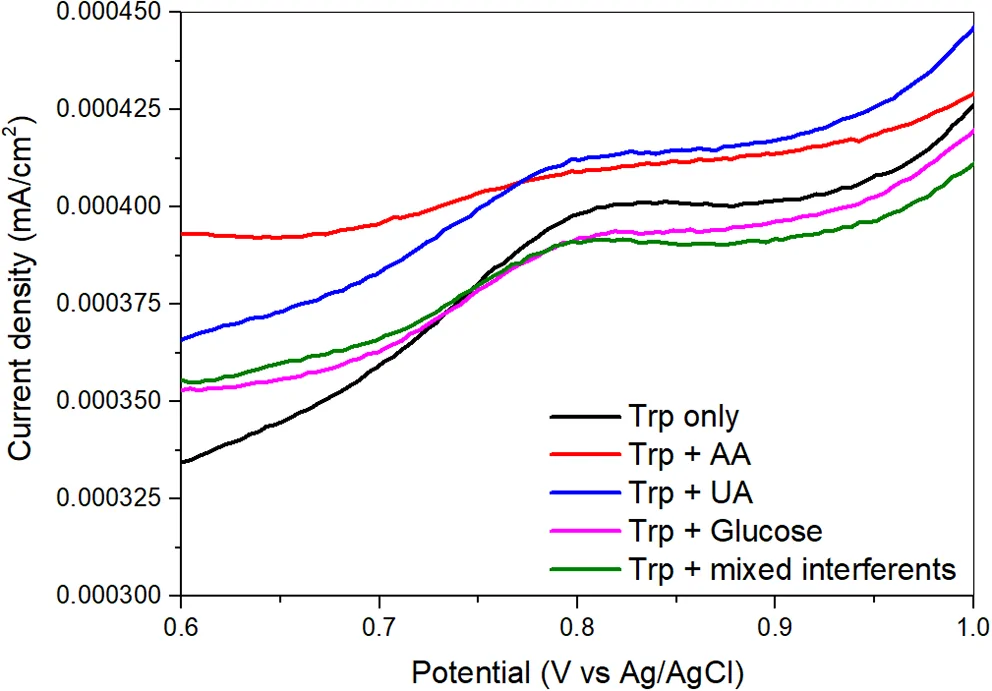
DPV responses of Ru–Pd/CNT@GCE toward tryptophan (Trp) in the absence and presence of ascorbic acid (AA), uric acid (UA), glucose (Glu), and mixed interferents in 0.1 M PBS (pH 7.0).

The relative response was calculated using the ratio of the Trp peak current in the presence of each interferent to that of Trp alone, which was taken as 100%. As summarized in Table 2, the relative responses were 102.5%, 103.3%, 98.5%, and 100.0% for Trp + AA, Trp + UA, Trp + Glu, and Trp + mixed interferents, respectively. These values remained within the range of 98.5–103.3%, indicating that the tested interferents caused only minor changes in the Trp signal. Therefore, the Ru–Pd/CNT@GCE sensor exhibited acceptable anti-interference performance toward Trp detection under the tested conditions.

**Table 2:** Relative Response of Ru–Pd/CNT@GCE toward Trp in the Presence of Biological Interferents

sample	peak current	relative response (%)
Trp	0.000400	100.0
Trp + AA	0.000410	102.5
Trp + UA	0.000413	103.3
Trp + Glu	0.000394	98.5
Trp + mixed interferents	0.000400	100.0

#### 3.2.4 Effect of Tryptophan Concentration

The influence of the tryptophan concentration on the electrochemical response of Ru–Pd/CNT@GCE was investigated by CV. CV curves were recorded in 0.1 M phosphate-buffered saline (PBS) containing tryptophan concentrations of 0, 5, 10, and 20 mM, as shown in Figure 11. In the absence of tryptophan, the Ru–Pd/CNT@GCE electrode exhibits a relatively low background current, whereas the introduction of tryptophan leads to a pronounced increase in the anodic current response. As the tryptophan concentration increases from 5 to 20 mM, an overall enhancement in the oxidation current density is observed (3.01–5.23 mA/cm^2^), indicating a concentration-dependent electrooxidation process. The fact that the current response has increased is a measure that this is due to the greater availability of tryptophan molecules on the electrode surface, and therefore, more electrochemical oxidation events occur. This tendency proves that the Ru–Pd/CNT@GCE sensor functions as an efficient electrocatalytic activity on the tryptophan oxidation and is sensitive to the alteration of the analyte concentration. The oxidation current did not show a strictly linear increase, particularly at high concentrations, which may be attributed to surface fouling/adsorption effects and mass transport limitations at the electrode surface.^61^ Such concentration-dependent behavior is essential for quantitative sensing applications and provides a strong basis for the subsequent analytical evaluation of the sensor using more sensitive techniques, such as DPV.^62^

**Figure 11:**
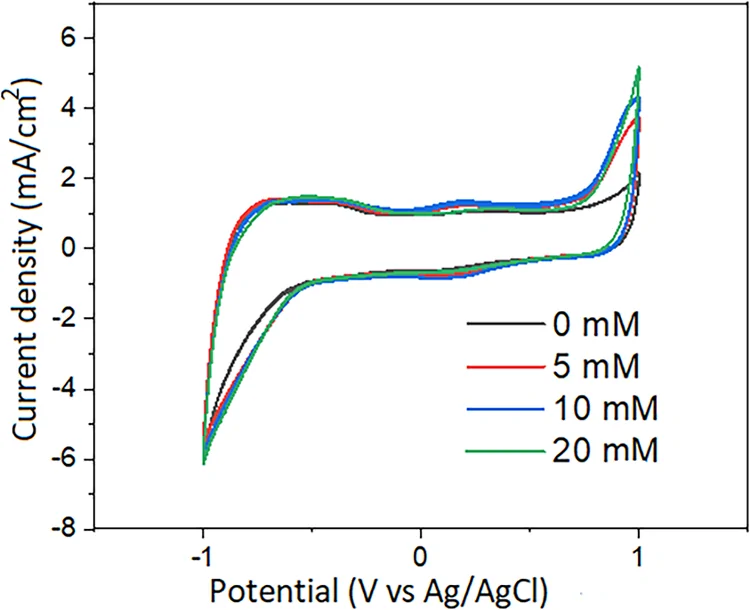
Cyclic voltammetric responses at different tryptophan concentrations recorded at a scan rate of 100 mV/s using the Ru–Pd/CNT@GCE sensor.

#### 3.2.5 Effect of Scan Rate and Kinetic Analysis

The effect of the scan rate on the electrochemical oxidation behavior of tryptophan at the Ru–Pd/CNT@GCE was investigated by CV. CV measurements were recorded in 0.1 M PBS containing 5 mM tryptophan over a scan rate range of 10–500 mV/s, as shown in Figure 12. As the scan rate increased, a gradual and systematic enhancement in the anodic oxidation current was observed, indicating accelerated electrochemical kinetics at higher scan rates. The growing current density as the scan rate increases is indicative of a mass-transport-controlled electrooxidation process of tryptophan.^63^ This tendency may be explained by the fact that with the diffusion layers moving toward the electrode surface, more molecules of tryptophan are able to pass through the diffusion layers and access the active sites as the potential sweeps increase. Good electrochemical stability and reproducibility of the Ru–Pd/CNT@GCE sensor are further ensured by the clearly defined voltammetric profiles that were obtained at various scan rates. To further demonstrate that the electrooxidation process is kinetic, the correlation between the logarithm of the maximum current density and the logarithm of the scan rate was plotted (the inset of Figure 12). The correlation coefficient (*R* ^2^ = 0.99) was also found to be high, indicating a diffusion-controlled electrochemical process. This outcome shows that the Ru–Pd/CNT@GCE sensor offers efficient charge-transfer mechanisms and positive diffusion properties that are needed to achieve stable and sensitive electrochemical detection of tryptophan.

**Figure 12:**
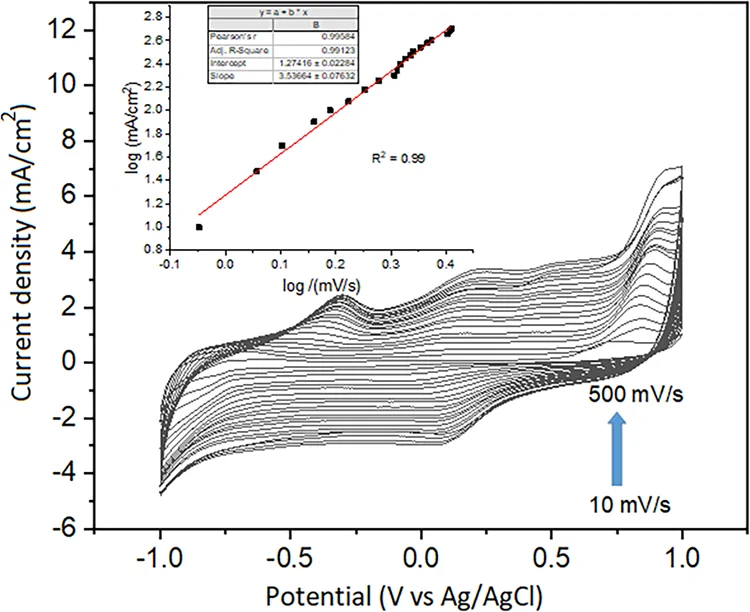
Effect of scan rate on the cyclic voltammetric response of the Ru–Pd/CNT@GCE sensor in the presence of 5 mM tryptophan in 0.1 M PBS.

#### 3.2.6 Quantitative Determination of Tryptophan

The electrochemical sensing characteristics of Ru–Pd/CNT@GCE were measured quantitatively using DPV to detect tryptophan. Figure 13a shows the DPV responses of the different tryptophan concentrations recorded in 0.1 M phosphate-buffered saline (PBS, pH 7.0). An oxidation peak is clearly seen, and with the rise in concentration of tryptophan over a broad concentration range of 1 to 1200 μM, there is a progressive increase in the intensity of the current, suggesting definite concentration-dependent oxidation of tryptophan. The graphical representation of peak current versus tryptophan concentration (Figure 13b) has a good linear relationship within the range of study with a regression equation of *y* = 0.0023*x* + 3.1104 and a correlation coefficient of *R* ^2^ = 0.9643. The sensitivity of the Ru–Pd/CNT@GCE sensor was estimated from the slope of the calibration curve, and the LOD was calculated using the equation LOD = 3.3σ/S, where σ (3.485 × 10^–5^) is the standard deviation of the intercept obtained from the calibration curve and S (0.0023) is the slope of the calibration curve. Based on this calculation, the LOD was determined to be 0.05 μM. Further, the sensor exhibits a wide LDR that ranges between 1 and 1200 μM, which is beneficial for analytical detection under controlled experimental conditions. The enhanced electrochemical performance of the Ru–Pd/CNT electrode can be attributed to the combined contribution of Ru–Pd bimetallic species and the conductive CNT support. The XPS, temperature-programmed analyses, EIS, and DFT results suggest mixed surface states, metal–support interactions, improved interfacial charge transfer, and electronic redistribution within the Ru–Pd/CNT system, which collectively contribute to the improved tryptophan-sensing performance. These findings indicate that the Ru–Pd/CNT@GCE sensor has promising potential for sensitive quantitative tryptophan detection under controlled PBS conditions.

**Figure 13:**
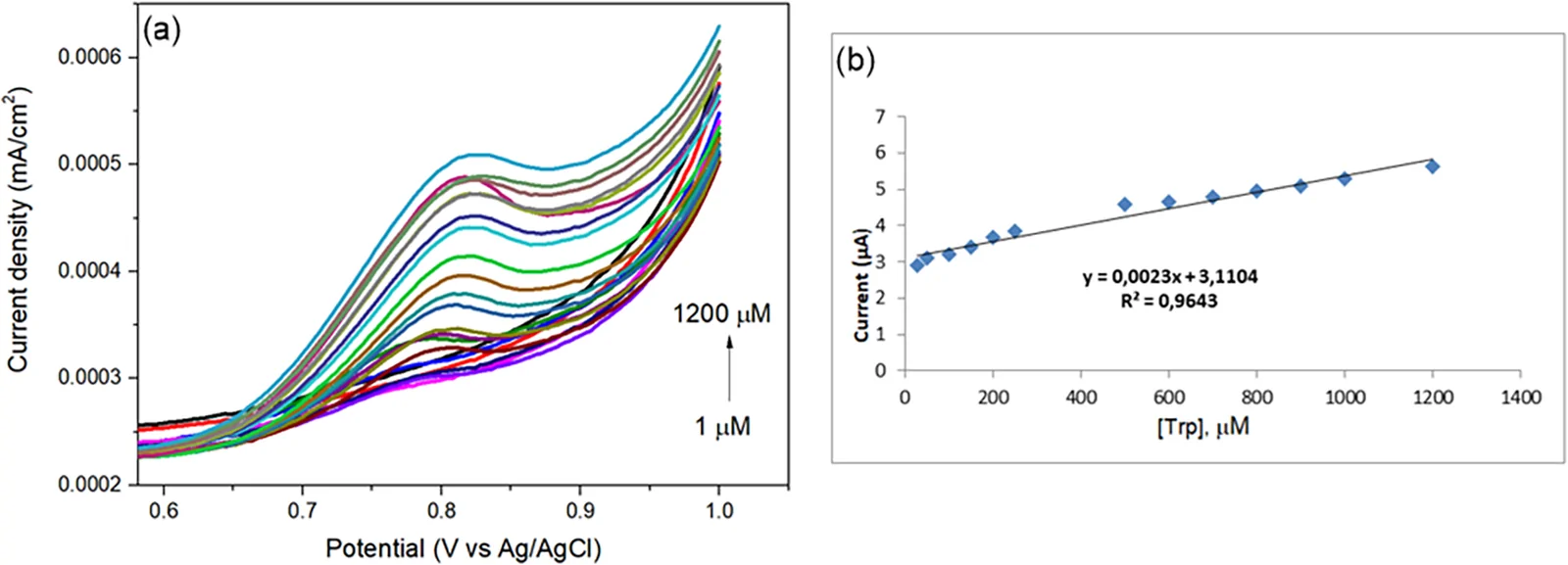
DPV responses of the Ru–Pd/CNT@GCE sensor toward different tryptophan concentrations in 0.1 M PBS (pH 7.0): (a) DPV curves and (b) corresponding calibration plot.

#### 3.2.7 EIS Measurements

EIS was used to study the interfacial charge-transfer mechanism, diffusion, and capacitive properties of Ru–Pd/CNT on GCE in the detection of tryptophan.^64^ The Nyquist plots obtained from the EIS measurements exhibit a semicircular region at high frequencies followed by a linear region at low frequencies, where the semicircle diameter corresponds to the charge-transfer resistance (*R*_ct_), while the linear region reflects diffusion-controlled processes at the electrode–electrolyte interface. Figure 14 presents the Nyquist plots of the Ru–Pd/CNT@GCE sensor obtained at different applied potentials in the range of 0.0–0.8 V in 0.1 M phosphate-buffered saline (PBS, pH 7.0) containing 5 mM tryptophan. With increasing applied potential, the diameter of the semicircle decreases gradually, and this corresponds to a decrease in the charge-transfer resistance and improved electron-transfer kinetics. The minimum *R*_ct_ was found at an applied potential of 0.8 V, which corresponds to the most favorable conditions for interfacial charge transfer during the oxidation of the amino acid, tryptophan. On the basis of the observed reduction in charge-transfer resistance with increasing applied potential, it was concluded that higher potentials facilitate charge transport across the Ru–Pd/CNT@GCE interface more efficiently. This behavior is due to the synergistic effect between the Ru–Pd bimetallic species and the highly conducting, large surface area of the CNT support, which rapidly transfers electrons and reduces the interfacial resistance. The electrochemical activity obtained from EIS is in good agreement with the results obtained in the CV and DPV modes, further proving the higher electrochemical activity of the Ru–Pd/CNT@GCE sensor. Therefore, 0.8 V was chosen as the optimum potential because the lowest charge-transfer resistance was obtained at this potential, enabling sensitive and efficient electrochemical detection of tryptophan.

**Figure 14:**
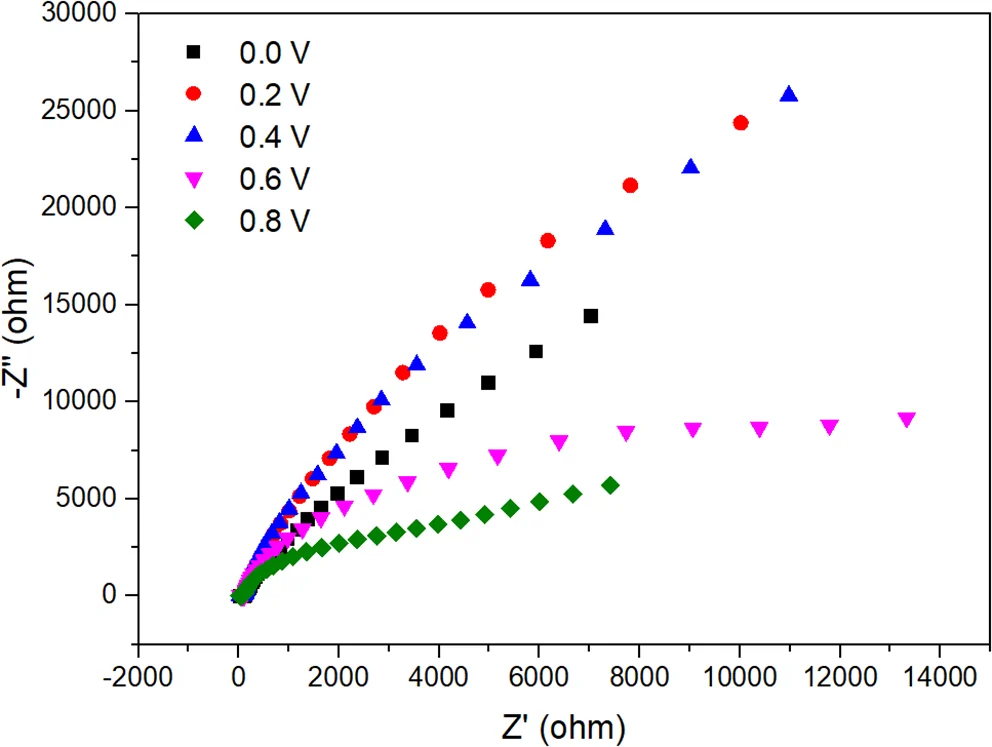
Nyquist plots obtained from EIS measurements of the Ru–Pd/CNT@GCE sensor in 0.1 M PBS (pH 7.0) containing 5 mM tryptophan at different applied potentials.

#### 3.2.8 Electroactive Surface Area Evaluation

The ECSA of bare GCE, CNT@GCE, Pd/CNT@GCE, Ru/CNT@GCE, and Ru–Pd/CNT@GCE was evaluated using the Randles–Sevcik method to compare the accessible electroactive sites of the different electrodes.^65^ CV measurements were recorded in 5 mM K_3_[Fe­(CN)_6_] containing 0.1 M KCl at scan rates of 20, 40, 60, 80, and 100 mV/s. As shown in Figure 15a–e, the anodic peak current increased with increasing scan rate for all electrodes, indicating diffusion-controlled redox behavior of the ferricyanide probe. The anodic peak current (*I*_pa_) was extracted from each CV curve and plotted against the square root of the scan rate (ν^1/2^), as shown in Figure 15f.

**Figure 15:**
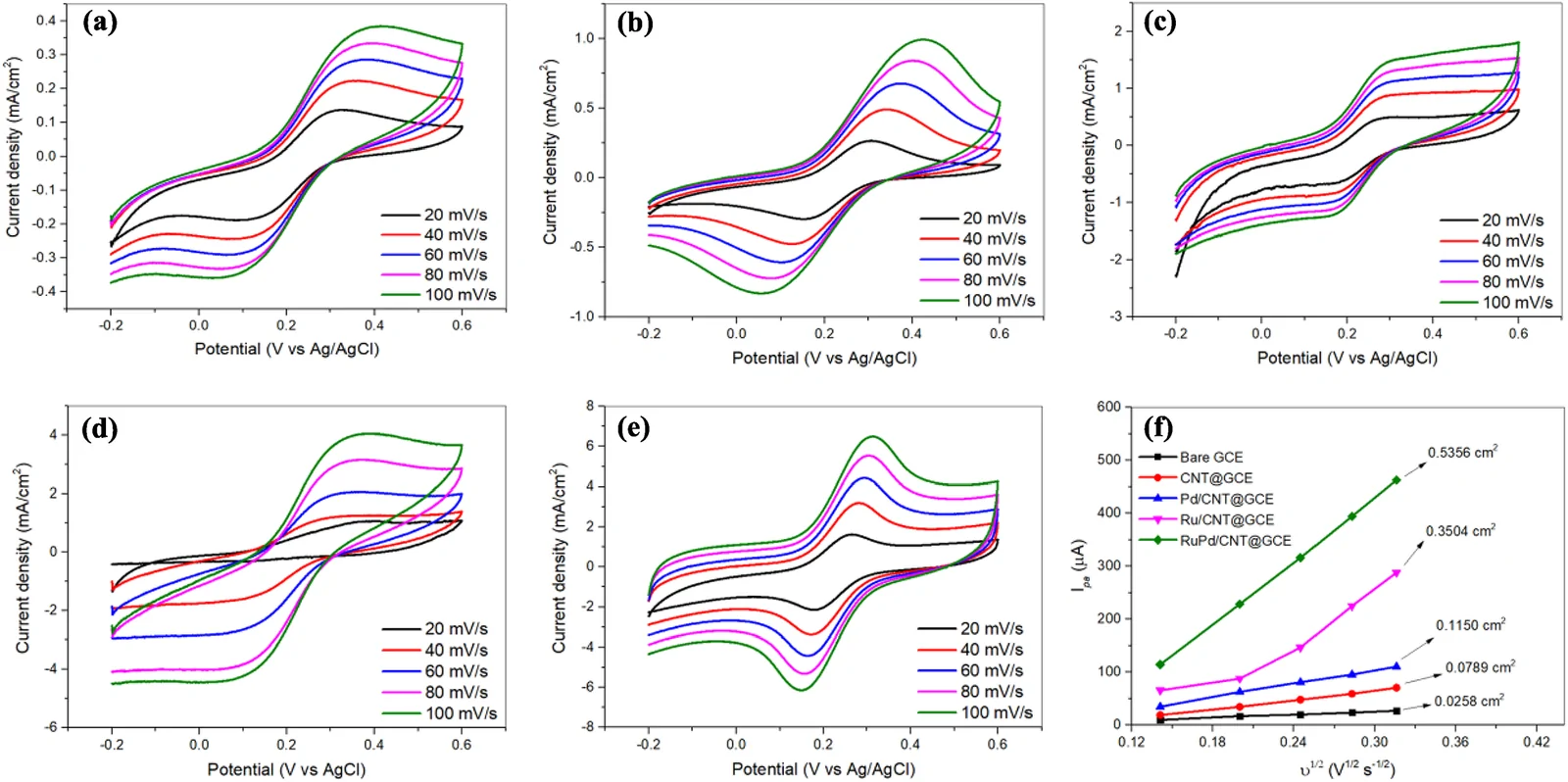
CV curves of (a) GCE, (b) CNT@GCE, (c) Pd/CNT@GCE, (d) Ru/CNT@GCE, and (e) Ru–Pd/CNT@GCE at different scan rates in 5 mM K_3_[Fe­(CN)_6_]/0.1 M KCl; (f) *I*_pa_ vs ν^1/2^ plots for ECSA calculation.

The ECSA was calculated using the Randles–Sevcik equation

$$I_{p}=2.69\times 10^{5}n^{3/2}{\mathrm{A D}}^{1/2}{\mathrm{C \nu}}^{1/2}$$

where *I*_p_ is the anodic peak current, *n* is the number of transferred electrons, *A* is the electroactive surface area, *D* is the diffusion coefficient of K_3_[Fe­(CN)_6_], *C* is the concentration of K_3_[Fe­(CN)_6_], and ν is the scan rate.^66^ Based on the slopes of the *I*_pa_ versus ν^1/2^ plots in Figure 15f, the calculated ECSA values were 0.0258, 0.0789, 0.1150, 0.3504, and 0.5356 cm^2^ for GCE, CNT@GCE, Pd/CNT@GCE, Ru/CNT@GCE, and Ru–Pd/CNT@GCE, respectively. The ECSA followed the order GCE < CNT@GCE < Pd/CNT@GCE < Ru/CNT@GCE < Ru–Pd/CNT@GCE, indicating that CNT modification and subsequent metal decoration increased the accessible electroactive surface area. The highest ECSA value obtained for Ru–Pd/CNT@GCE suggests that the bimetallic Ru–Pd/CNT structure provides the largest number of accessible electroactive sites among the investigated electrodes. This enhanced electroactive surface area can facilitate interfacial electron transfer and contribute to the improved electrochemical sensing performance of Ru–Pd/CNT@GCE toward tryptophan oxidation.

#### 3.2.9 Short-Term Chronoamperometric Stability

The operational stability of the Ru–Pd/CNT@GCE sensor was also checked using chronoamperometry in 0.1 M PBS containing 5 mM tryptophan. At the beginning of the measurement, a rapid change in current was seen, as shown in Figure 16, which may be related to rapid electrode/electrolyte interface stabilization processes. This initial transient phase was followed by a quasi-stable phase, where the current remained relatively steady for up to 2600 s. This finding revealed that the Ru–Pd/CNT@GCE sensor shows good short-term chronoamperometric stability under the conditions used in this study.

**Figure 16:**
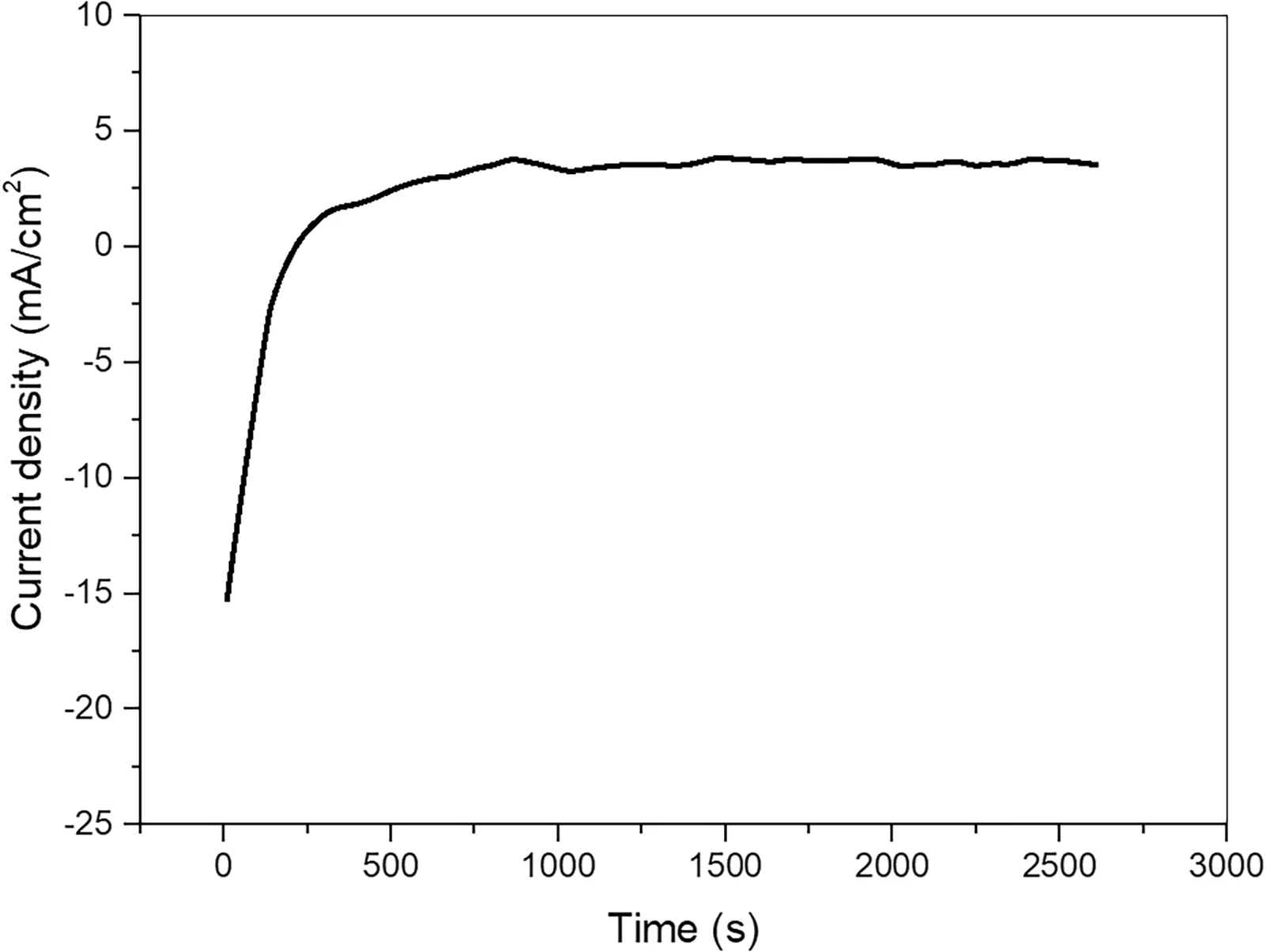
Chronoamperometric response of Ru–Pd/CNT@GCE in 0.1 M PBS containing 5 mM tryptophan at an applied potential of 0.8 V for 2600 s.

### 3.3 Comparative Evaluation of Sensor Performance

The performance of the Ru–Pd/CNT@GCE sensor in tryptophan detection was evaluated by comparison with previously reported sensors, as summarized in Table 3. According to the literature, a broad range of sensing platforms and detection strategies has been reported, including colorimetric,^67^ fluorescence,^68^ and electrochemical methods such as amperometry,^69^ CV,^70^ linear sweep voltammetry (LSV),^71^ square wave voltammetry (SWV),^72^ and DPV.^73–75^ These methods exhibit different sensitivities and linear working ranges. Colorimetric sensors, such as 4-DPD-Ag NPs, generally present relatively narrow linear dynamic ranges (LDRs) and higher limits of detection (LODs), making them less suitable for low-concentration tryptophan analysis. Fluorescence-based sensors generally provide better sensitivity, as reflected by their lower LOD values, although their concentration range is often limited. Electrochemical sensors are typically advantageous because of their high sensitivity and operational simplicity; however, their performance depends strongly on the electrode material and the measurement technique used. Among the electrochemical techniques, DPV-based sensors have been widely used because of their high sensitivity. However, some of the reported DPV sensors, including BDD and Nafion/TiO_2_-GR/GCE, have either high LODs or small LDRs. Conversely, the Ru–Pd/CNT@GCE sensor in this work has a wide LDR of 1–1200 μM with a low detection limit of 0.05 μM, which is better than most of the previously reported tryptophan sensors in terms of sensitivity and analytical range. This high performance may be attributed to the combined contribution of Ru–Pd bimetallic species and the high conductivity of the CNT support, which together facilitate efficient electron transfer and promote the electrooxidation of tryptophan. This combination allows sensitive detection over a wide concentration range. The proposed sensor is especially promising for practical applications requiring low detection limits together with a wide analytical range.

**Table 3:** Comparison of the Analytical Performance Parameters, Including Detection Method, LDR, and LOD, of the Ru–Pd/CNT@GCE Sensor with Previously Reported Sensors for Tryptophan Detection

sensor	method	LDR (μM)	LOD (μM)	refs
4-DPD-Ag NPs	Colorimetric	75–500	20	^67^
CCS	Fluorescence	0–60	0.31	^68^
TiO_2_-MWNT/GCE	Amperometry	1–150	0.52	^69^
4-ABA/GCE	CV	1–100	0.2	^70^
CuNPs@PoPD/GCE	LSV	1–9.8	1.08	^71^
GR	SWV	0.3–40	0.1	^72^
BDD	DPV	20–1000	10	^73^
Nafion/TiO_2_-GR/GCE	DPV	5–140	0.7	^74^
PGMCNTPS	DPV	20–100	0.42	^75^
Ru–Pd/CNT@GCE	DPV	1–1200	0.05	This work

### 3.4 DFT Analysis of CNT-Based Catalysts

DFT calculations were carried out to investigate the electronic structures of pristine CNT, Pd/CNT, Ru/CNT, and Ru–Pd/CNT and to provide theoretical support for the experimentally observed sensing performance. It should be emphasized that the calculated CNT structure represents a simplified local CNT-wall model of the experimentally used MWCNT support, not a full multiwalled CNT structure with defined chirality. Optimized geometries, frontier molecular orbitals, global reactivity descriptors, MEP, LOL, and ELF were analyzed to establish a clear structure–property-performance correlation. Figure 17 shows the DFT-optimized structures of (a) CNT, (b) Pd/CNT, (c) Ru/CNT, and (d) Ru–Pd/CNT. Figure 17a shows that the pristine CNT maintains its cylindrical geometry with a regular sp^2^-hybridized carbon structure. The optimized structure indicates that CNT is structurally stable and retains its extended carbon framework after geometry optimization. This confirms that CNT mainly acts as a structurally robust and conductive support material.^76^ In the Pd/CNT and Ru/CNT systems (Figure 17b,c), the individual Pd and Ru atoms were stabilized on the CNT surface after geometry optimization, indicating the formation of metal–support interactions. Compared with pristine CNT, the introduction of Pd or Ru creates additional metal-centered regions on the CNT surface, which may serve as electrochemically active sites for tryptophan oxidation. In the Ru–Pd/CNT system (Figure 17d), the optimized structure shows the simultaneous stabilization of both Ru and Pd atoms on the CNT surface. The presence of both metals generates additional electronically active regions and provides a bimetallic surface environment, which is absent in pristine CNT and the monometallic systems. These regions are expected to contribute to the electrochemical sensing process, while the CNT backbone provides an efficient pathway for electron transport.^77,78^ Therefore, the Ru–Pd/CNT structure combines the conductive nature of CNT with the electronic contribution of Ru and Pd sites, which is beneficial for improving the charge-transfer behavior and sensing performance of the electrode.

**Figure 17:**
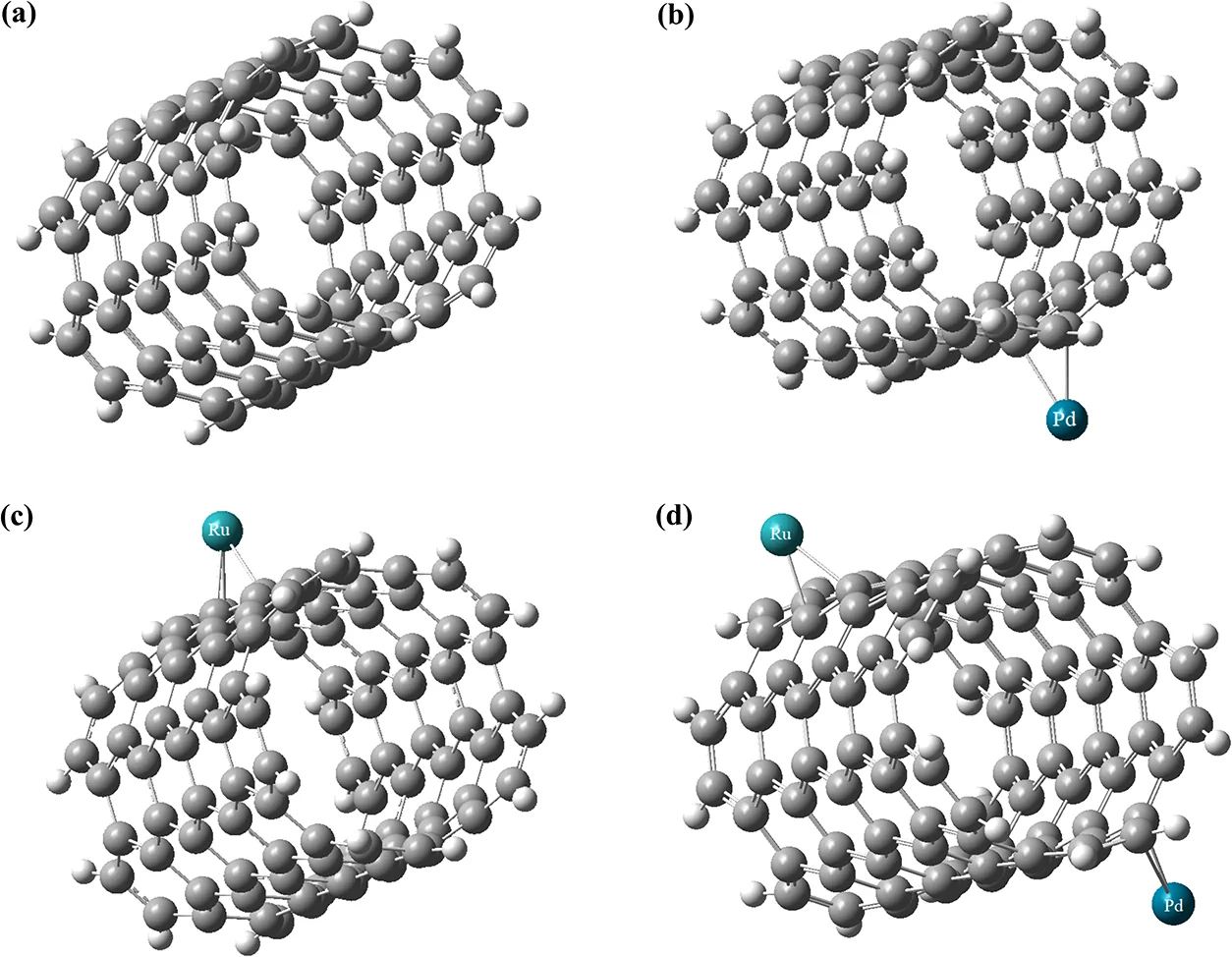
DFT-optimized geometries of (a) CNT, (b) Pd/CNT, (c) Ru/CNT, and (d) Ru–Pd/CNT.

Figure 18 represents the frontier molecular orbital distributions and energy gaps of the CNT, Pd/CNT, Ru/CNT, and Ru–Pd/CNT. In the case of pristine CNT (Figure 18a), the HOMO and LUMO orbitals are predominantly concentrated on the carbon structure. The HOMO–LUMO energy difference is calculated to be 7.498 eV, which is relatively high. This sizable energy gap indicates lower electronic reactivity and suggests that pristine CNT mainly acts as a conductive support rather than as a highly active electron-transfer site. After metal decoration, clear changes in the frontier orbital distribution and energy gaps were observed. The introduction of Pd at the CNT surface caused a change in the HOMO and LUMO distributions in Pd/CNT (as shown in Figure 18b) and lowered the energy gap to 5.453 eV. A larger decrease in energy gap to 1.028 eV was found in Ru/CNT CNT (Figure 18c) in the present work, which further supports the electronic effect of Ru decoration on the surface of the CNT. When Ru–Pd decoration was performed, a more significant electronic structure difference was observed. The HOMO and LUMO orbitals in Ru–Pd/CNT (Figure 18d) appear to be confined to the Ru and Pd atoms, as well as the adjacent C atoms. The HOMO–LUMO energy gap dropped significantly to 0.466 eV, suggesting that the bimetallic Ru–Pd system has the most electronically activated CNT surface among the systems investigated. The energy gap was ranked as CNT > Pd/CNT > Ru/CNT > Ru–Pd/CNT, thus demonstrating that the incorporation of Ru and Pd together allows for better electronic redistribution and charge transfer. Reduction of the energy gap can facilitate electron transfer from the surface of the electrode to the analyte molecule, which is essential for an effective electrochemical detection. The co-introduction of Ru and Pd has been found to have a significant effect on electronically activating the CNT surface and promoting an increased response toward the oxidation of the amino acid tryptophan.

**Figure 18:**
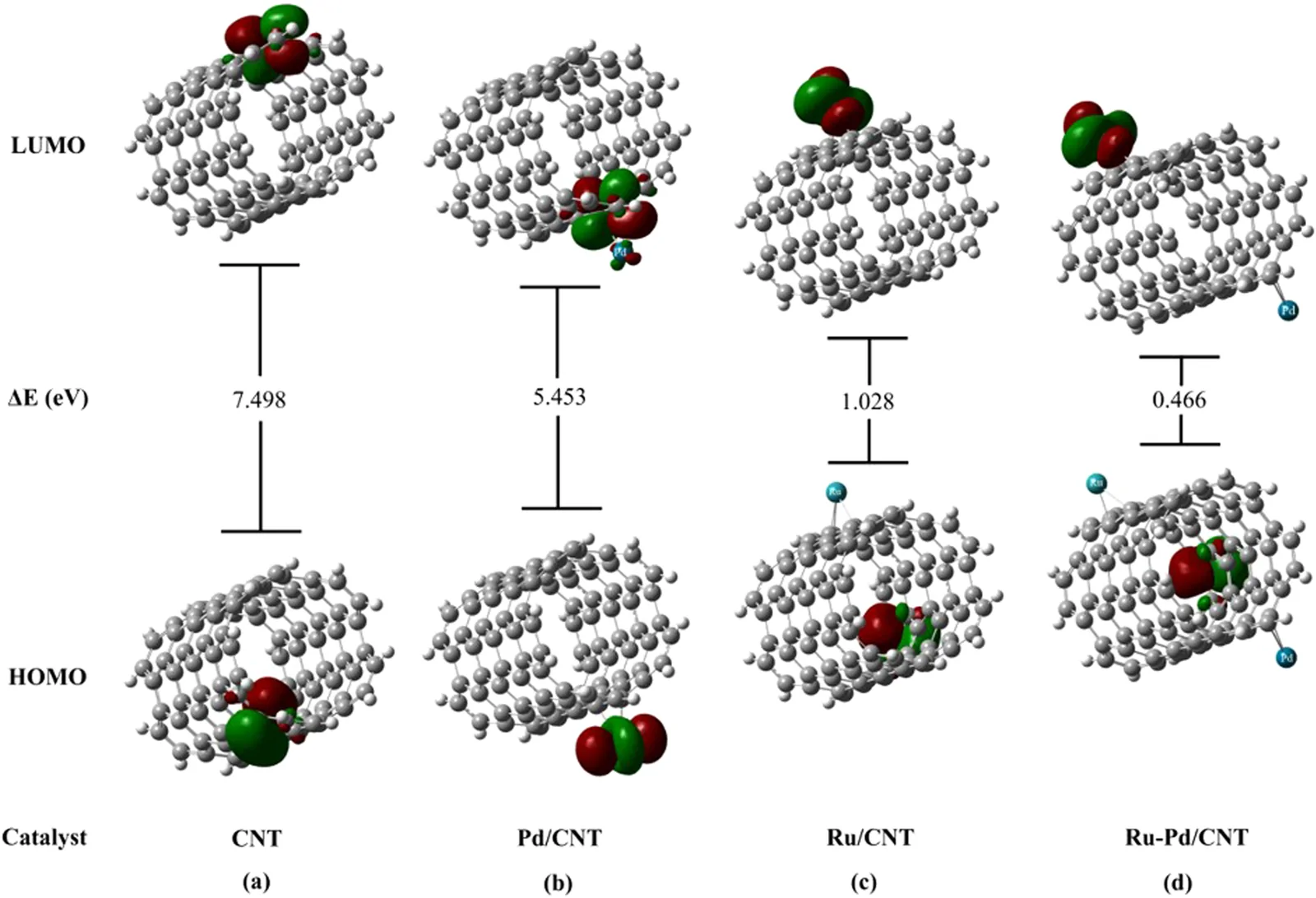
Frontier molecular orbital distributions and HOMO–LUMO energy gaps of (a) pristine CNT, (b) Pd/CNT, (c) Ru/CNT, and (d) Ru–Pd/CNT.

The effect of decoration with monometallic and bimetallic entities (CNT, Pd/CNT, Ru/CNT, and Ru–Pd/CNT) on the electronic activity relevant to electrochemical sensing can be readily determined by making a direct comparison of the frontier molecular orbital energies and global reactivity descriptors in Table 4. Pristine CNT possesses a low HOMO energy and a positive LUMO energy, meaning that it is not a good donor or acceptor. The electronic structure showed that although CNTs are highly conductive, they do not have high inherent electrocatalytic activity, but rather are used as a conductive support. After the decoration process, the energy gap between the HOMO and LUMO was reduced from 7.498 eV for CNT to 5.453 eV for Pd/CNT, suggesting that the energy gap was partially reduced after decoration. For Ru/CNT, a clearly larger decrease was observed, with the energy gap reducing to 1.028 eV, indicating increased involvement of the Ru sites in the charge-transfer process. In the case of Ru–Pd/CNT, the improvement in the frontier orbital energies is the most pronounced. The LUMO energy is lowered to −2.942 eV, significantly improving the energetics of the electron-accepting properties of this system, while the HOMO energy level is elevated to −3.408 eV, improving the energetics of the electron-donation properties. Efficient electron exchange between the electrode surface and analyte molecules is essential in electrochemical oxidation reactions, and this dual enhancement of donor–acceptor ability is crucial. The HOMO–LUMO energy gap decreases in the following order: CNT > Pd/CNT > Ru/CNT > Ru–Pd/CNT, from 7.498 eV for pristine CNT to 0.466 eV for Ru–Pd/CNT. This trend is consistent with the results observed for the most electronically activated CNT surface, namely, the Ru–Pd-decorated surface, among the investigated systems. It should be noted that the change in frontier orbital energies is substantial, but the chemical potential of CNT (−3.078 eV) and Ru–Pd/CNT (−3.175 eV) remains fairly close. This indicates that the increased electronic activity of Ru–Pd/CNT is not linked to electronic instability, but instead to more favorable electronic redistribution. At the same time, the chemical hardness (η) of CNT decreases significantly from 3.749 to 0.233 eV, signifying a transition from a rigid electronic structure to a more polarizable and reactive structure. In contrast, the electrophilicity index (ω) increases from 1.26 eV for CNT to 21.64 eV for Ru–Pd/CNT, signifying that the bimetallic catalyst has an enhanced electron-accepting ability. Thus, Ru–Pd/CNT has the lowest energy gap and highest electrophilicity index compared with Pd/CNT and Ru/CNT, indicating that the combined electronic effect of Ru and Pd on the CNT surface is beneficial. Our comparison implies that Ru–Pd decoration transforms CNT from a stable but weakly reactive conductive material into a highly active surface for the electrocatalytic oxidation of tryptophan.

**Table 4:** Calculated Frontier Molecular Orbital Energies and Global Reactivity Descriptors for CNT, Pd/CNT, Ru/CNT, and Ru–Pd/CNT Catalysts

catalyst	*E*_HOMO_ (eV)	*E*_LUMO_ (eV)	Δ*E* (eV)	μ (eV)	η (eV)	ω (eV)
CNT	–6.827	0.671	7.498	–3.078	3.749	1.26
Pd/CNT	–6.094	–0.641	5.453	–3.368	2.726	2.08
Ru/CNT	–4.419	–3.391	1.028	–3.905	0.514	14.84
Ru–Pd/CNT	–3.408	–2.942	0.466	–3.175	0.233	21.64

Figure 19 presents the MEP maps of pristine CNT, Pd/CNT, Ru/CNT, and Ru–Pd/CNT, which display the surface charge distribution in terms of electrostatic potential values.^79^ The blue regions have positive electrostatic potential values, while the red-yellow regions have negative electrostatic potential values.^80,81^ The MEP values of pristine CNT (Figure 19a) range from −4.363 × 10^–2^ to +4.363 × 10^–2^ a.u. The blue areas are regions with reduced electron density, whereas the yellow areas around the carbon framework are regions with increased electron density. The distribution is fairly uniform, indicating low surface polarization. After metal decoration, the potential distribution becomes more complex. For Pd/CNT in Figure 19b, the MEP range is from −3.565 × 10^–2^ to +3.565 × 10^–2^ a.u., indicating alteration of the local electrostatic environment around the metal site and neighboring carbon atoms by the Pd atom. The MEP values of Ru/CNT shown in Figure 19c are between −4.510 × 10^–2^ and +4.510 × 10^–2^ a.u., indicating higher surface polarization around the Ru site in Ru/CNT. These changes suggest modification of the charge distribution of CNT and the formation of additional electronically active regions as a result of monometallic Pd and Ru decoration. For Ru–Pd/CNT (Figure 19d), the MEP values range from −3.660 × 10^–2^ to +3.660 × 10^–2^ a.u. The combined presence of Ru and Pd brings about a more modified electrostatic environment than pristine CNT and the monometallic systems. Moderate positively charged electrostatic potential is shown as light-blue regions around the Ru and Pd atoms, whereas yellow regions around the carbon framework indicate the electron-rich character of the CNT backbone. This shift in electrostatic potential suggests that bimetallic decoration with Ru–Pd creates a heterogeneous surface, which enhances the local electrostatic conditions for interactions between the analyte and the surface. This electrostatic configuration can be useful for electrochemical sensing, since these regions around the metal atoms could facilitate the interaction with tryptophan molecules, while the CNT framework could assist in efficient charge transport during electrooxidation.

**Figure 19:**
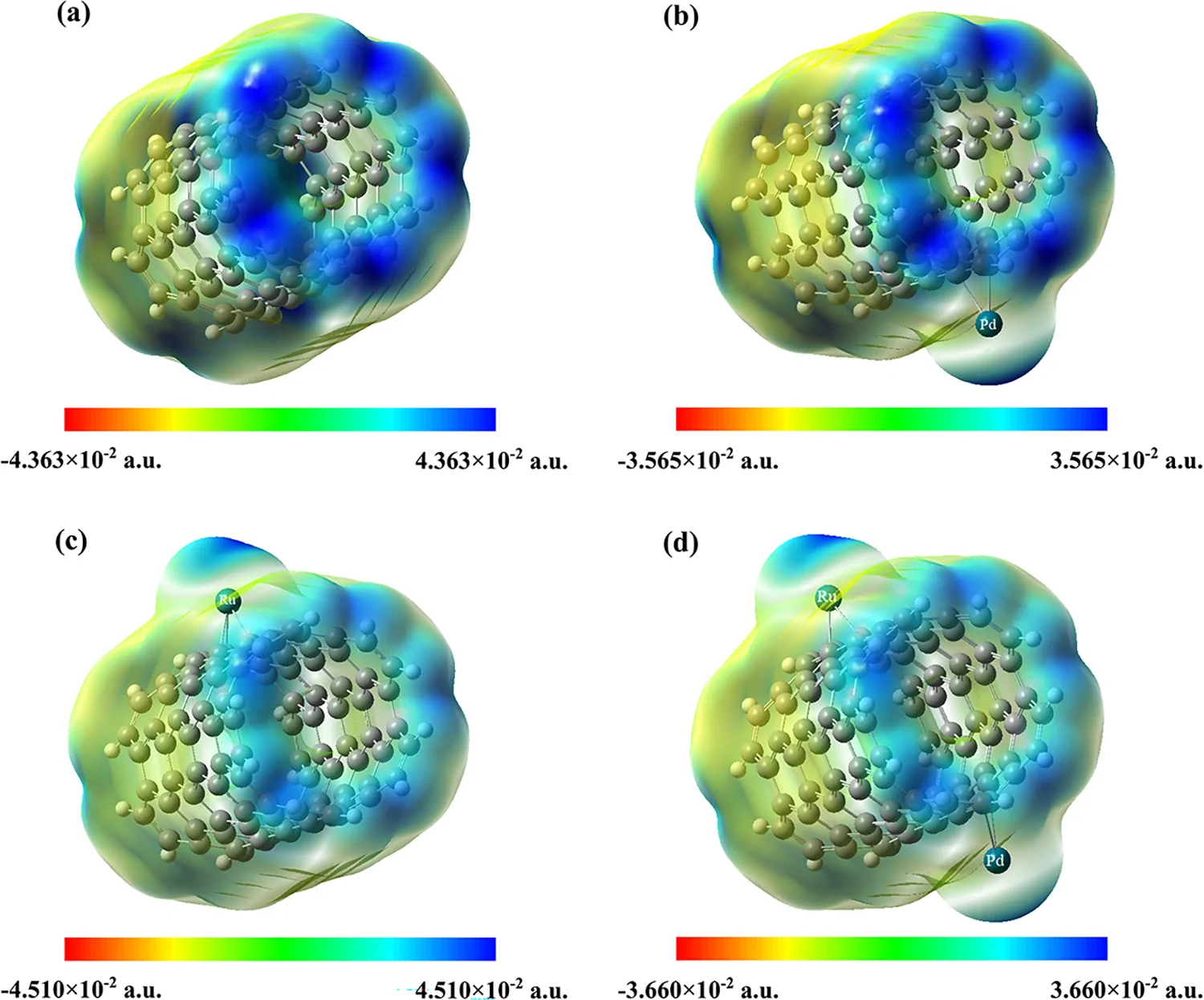
MEP maps of (a) pristine CNT, (b) Pd/CNT, (c) Ru/CNT, and (d) Ru–Pd/CNT.

ELF analysis provides insight into the degree of electron localization in a system and helps distinguish highly localized electronic regions, which are commonly associated with bonding or active sites, from delocalized regions that contribute to charge transport.^82,83^ The ELF distributions of (a) pristine CNT, (b) Pd/CNT, (c) Ru/CNT, and (d) Ru–Pd/CNT are shown in Figure 20. For pristine CNT (Figure 20a), the ELF map mainly reflects the electron localization associated with the *sp* ^2^-hybridized carbon framework. The orange-yellow regions correspond to localized electrons related to C–C covalent bonding, whereas the blue regions indicate more delocalized electronic areas.^84,85^ This distribution suggests that pristine CNT possesses a stable carbon framework but limited localized active regions for direct analyte interactions. After metal decoration, the ELF distribution became more modified. In Pd/CNT (Figure 20b), localized ELF regions appear more clearly along the CNT surface, indicating electronic interaction between Pd and the neighboring carbon atoms. Similarly, Ru/CNT (Figure 20c) shows enhanced electron localization in the metal-decorated region, suggesting stronger metal–support interaction and the creation of additional electronically active sites. These results indicate that monometallic Pd and Ru decoration modifies the electron localization pattern of the CNT. For Ru–Pd/CNT (Figure 20d), the ELF distribution shows a more favorable combination of localized and delocalized electronic regions. Localized regions are present around the metal-decorated areas, while delocalized regions remain distributed over the CNT frameworks. This indicates that the Ru–Pd/CNT system can preserve the conductive character of CNT while introducing additional localized electronic regions associated with metal–support interactions. Such a balanced electron localization–delocalization pattern can facilitate interfacial charge transfer and provide theoretical support for the enhanced electrochemical sensing performance of the Ru–Pd/CNT electrode toward tryptophan oxidation.

**Figure 20:**
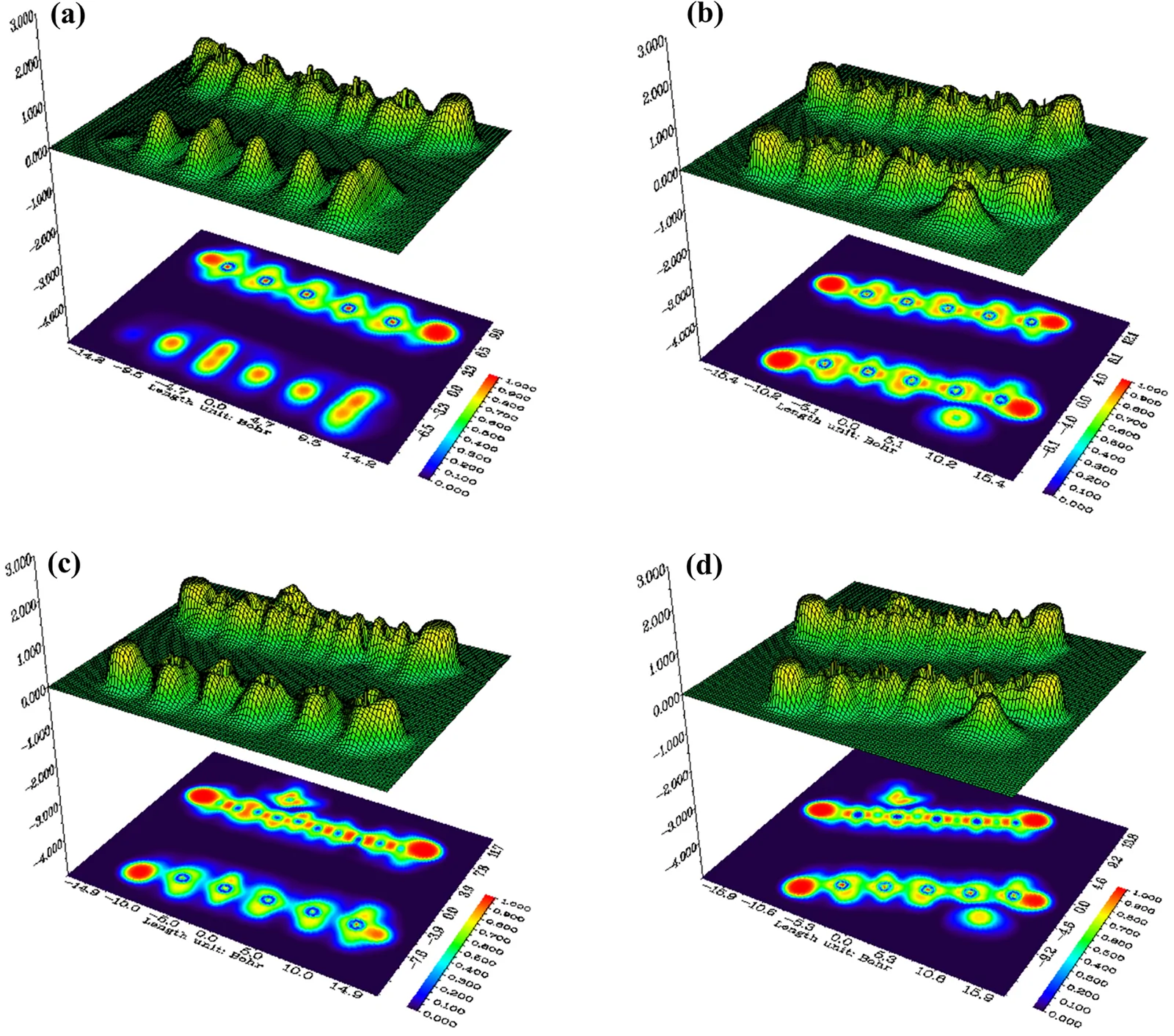
ELF distributions of (a) pristine CNT, (b) Pd/CNT, (c) Ru/CNT, and (d) Ru–Pd/CNT.

Figure 21 compares the LOL distributions of (a) pristine CNT, (b) Pd/CNT, (c) Ru/CNT, and (d) Ru–Pd/CNT. LOL analysis provides information on the spatial localization of electronic orbitals, where orange regions represent strongly localized electronic orbitals, yellow-green regions represent moderately localized electronic orbitals, and blue regions represent delocalized electronic orbitals.^86,87^ In the case of pristine CNT (Figure 21a), the LOL map is dominated by a blue background, indicating that delocalized electronic states are predominant over the CNT surface. Moderate orbital localization is observed along the carbon framework, while strongly localized regions appear in only limited areas. This distribution suggests that pristine CNTs mainly act as a conductive framework with limited localized active regions. After monometallic decoration, noticeable changes in the LOL distributions are observed. In Pd/CNT (Figure 21b), the Pd atom modifies the orbital distribution and generates more pronounced localized regions around the metal-decorated area and the neighboring carbon atoms. Similarly, Ru/CNT (Figure 21c) exhibits stronger localized orbital features around the Ru site, indicating enhanced metal–support interaction and the formation of additional electronically active regions. These results confirm that both Pd and Ru decoration alter the orbital distribution of CNT and introduce new active sites. In Ru–Pd/CNT (Figure 21d), the LOL distribution exhibits a more favorable localization pattern, in which localized orbital regions are associated with the Ru- and Pd-decorated sites, while the CNT framework still preserves delocalized conductive regions. Compared with pristine CNT and the monometallic systems, Ru–Pd/CNT shows a more balanced localization–delocalization pattern and improved orbital connectivity. This electronic arrangement may facilitate interfacial charge transfer and provide theoretical support for the enhanced electrochemical sensing performance of the Ru–Pd/CNT electrode toward tryptophan oxidation.

**Figure 21:**
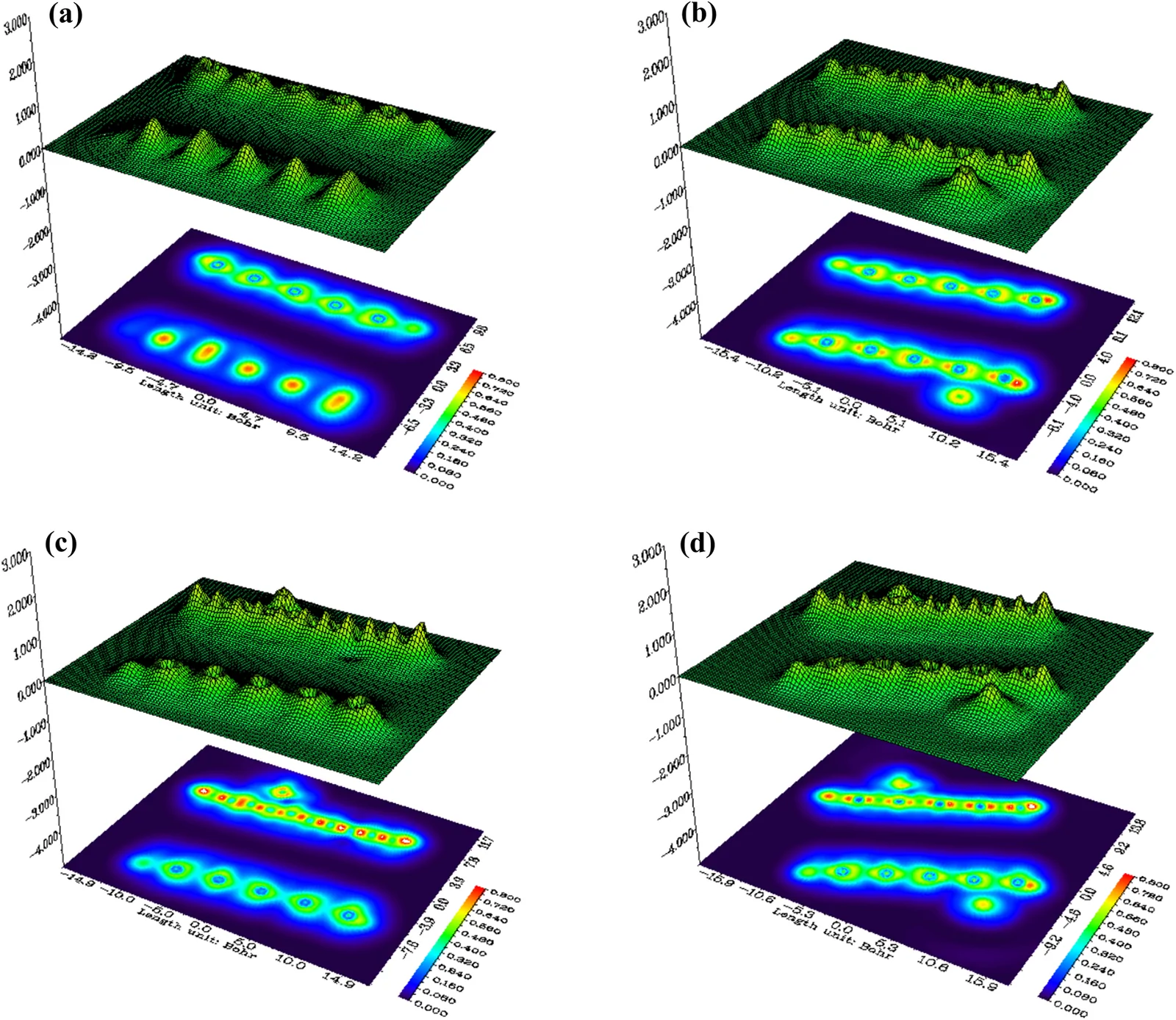
LOL distributions of (a) pristine CNT, (b) Pd/CNT, (c) Ru/CNT, and (d) Ru–Pd/CNT.

In summary, the DFT results suggest that metal decoration progressively activates the CNT surface, with the Ru–Pd/CNT system showing the most favorable combination of reduced HOMO–LUMO gap, modified surface polarization, and balanced electron localization–delocalization. These features support the experimentally observed improvement in charge-transfer behavior and sensing performance of the Ru–Pd/CNT@GCE electrode for tryptophan detection.

## 4 Conclusion

In this study, a highly sensitive electrochemical sensor for tryptophan detection was successfully developed using a bimetallic Ru–Pd/CNT. The Ru–Pd/CNT nanocomposite was synthesized via a facile NaBH_4_ reduction method, leading to the uniform dispersion of Ru and Pd nanoparticles on the CNT surface and strong metal–support interactions, as confirmed by comprehensive physicochemical characterization. The Ru–Pd/CNT@GCE, when used as a modifier on a GCE, showed high electrocatalytic activity toward tryptophan oxidation. The sensor exhibited a broad LDR of 1–1200 μM, a low detection limit of 0.05 μM, high sensitivity, and acceptable selectivity/anti-interference performance in the presence of the tested amino acids and common biological interferents under controlled PBS conditions. The short-term chronoamperometric response also indicated acceptable operational stability under the tested conditions. EIS also showed improved charge-transfer kinetics at the modified electrode interface. DFT calculations provided a clear theoretical explanation for the enhanced sensing performance. The marked reduction in the HOMO–LUMO energy gap after Ru–Pd decoration, together with favorable electron redistribution and strong metal–CNT electronic interactions, supports the enhanced electronic conductivity and charge-transfer efficiency observed experimentally. The results show that the bimetallic Ru–Pd species and conductive CNT support produce an effective and highly sensitive electrochemical sensing platform for tryptophan detection. To further prove the sensor’s applicability, future studies will involve validation in complex biological and food matrices, comparison with standard reference methods such as HPLC, as well as fouling mitigation, repeated use, interelectrode reproducibility, and long-term operational stability. The Ru–Pd/CNT@GCE sensor thus has great potential for sensitive tryptophan analysis under controlled analytical conditions and could be regarded as a promising bimetallic CNT-based material for the design of more complex electrochemical sensors.
